# A Piezo1/KLF15/IL-6 axis mediates immobilization-induced muscle atrophy

**DOI:** 10.1172/JCI154611

**Published:** 2022-05-16

**Authors:** Yu Hirata, Kazuhiro Nomura, Daisuke Kato, Yoshihisa Tachibana, Takahiro Niikura, Kana Uchiyama, Tetsuya Hosooka, Tomoaki Fukui, Keisuke Oe, Ryosuke Kuroda, Yuji Hara, Takahiro Adachi, Koji Shibasaki, Hiroaki Wake, Wataru Ogawa

**Affiliations:** 1 Division of Diabetes and Endocrinology, Department of Internal Medicine, Kobe University Graduate School of Medicine, Kobe, Japan.; 2 Department of Anatomy and Molecular Cell Biology, Nagoya University Graduate School of Medicine, Nagoya, Japan.; 3 Department of Physiology and Cell Biology,; 4 Department of Orthopaedic Surgery, and; 5 Division of Development of Advanced Therapy for Metabolic Disease, Department of Internal Medicine, Kobe University Graduate School of Medicine, Kobe, Japan.; 6 Laboratory of Nutritional Physiology, School of Food and Nutritional Sciences/Graduate School of Integrated Pharmaceutical and Nutritional Sciences and; 7 Department of Integrative Physiology, School of Pharmaceutical Sciences, University of Shizuoka, Shizuoka, Japan.; 8 Department of Precision Health, Medical Research Institute, Tokyo Medical and Dental University, Tokyo, Japan.; 9 Laboratory of Neurochemistry, Graduate School of Human Health Science, University of Nagasaki, Nagasaki, Japan.

**Keywords:** Metabolism, Muscle Biology, Calcium signaling, Skeletal muscle

## Abstract

Although immobility is a common cause of muscle atrophy, the mechanism underlying this causality is unclear. We here show that Krüppel-like factor 15 (KLF15) and IL-6 are upregulated in skeletal muscle of limb-immobilized mice and that mice with KLF15 deficiency in skeletal muscle or with systemic IL-6 deficiency are protected from immobility-induced muscle atrophy. A newly developed Ca^2+^ bioimaging revealed that the cytosolic Ca^2+^ concentration ([Ca^2+^]_i_) of skeletal muscle is reduced to below the basal level by immobilization, which is associated with the downregulation of Piezo1. Acute disruption of *Piezo1* in skeletal muscle induced *Klf15* and *Il6* expression as well as muscle atrophy, which was prevented by antibodies against IL-6. A role for the Piezo1/KLF15/IL-6 axis in immobility-induced muscle atrophy was validated in human samples. Our results thus uncover a paradigm for Ca^2+^ signaling in that a decrease in [Ca^2+^]_i_ from the basal level triggers a defined biological event.

## Introduction

A decline in skeletal muscle mass can lead to a variety of detrimental conditions and a consequent shortening of life (1). Although many pathological conditions — including neuromuscular, cardiovascular, metabolic, inflammatory, and malignant diseases — trigger muscle atrophy, a decrease in physical activity or immobility is one of the most common and clinically important causes of muscle loss. Immobility-induced muscle atrophy occurs in various settings, including neural paralysis, musculoskeletal disorders, bed rest, and aging, but its molecular mechanism has remained largely unknown.

The cytosolic concentration of Ca^2+^ ([Ca^2+^]_i_) is generally maintained low (in the tens of nanomolar range) under basal conditions but is increased by a factor of 10 to 100 in response to extracellular stimuli (2). The mechanisms by which [Ca^2+^]_i_ increases and triggers a variety of biological responses have been studied extensively (3). How the basal level of cytosolic Ca^2+^ is maintained in cells and whether a decline in this level might also trigger biological responses have been unclear, however. We now show that the Ca^2+^ channel Piezo1 in the cell membrane contributes to maintenance of the basal [Ca^2+^]_i_ in skeletal muscle, and that a decrease in this concentration from the basal level due to inhibition of Piezo1 triggers muscle atrophy associated with immobilization via a signaling pathway mediated by the transcription factor Krüppel-like factor 15 (KLF15) and the cytokine interleukin-6 (IL-6).

## Results

### Immobilization-induced skeletal muscle atrophy in mice is prevented by KLF15 deficiency.

**Figure 1:**
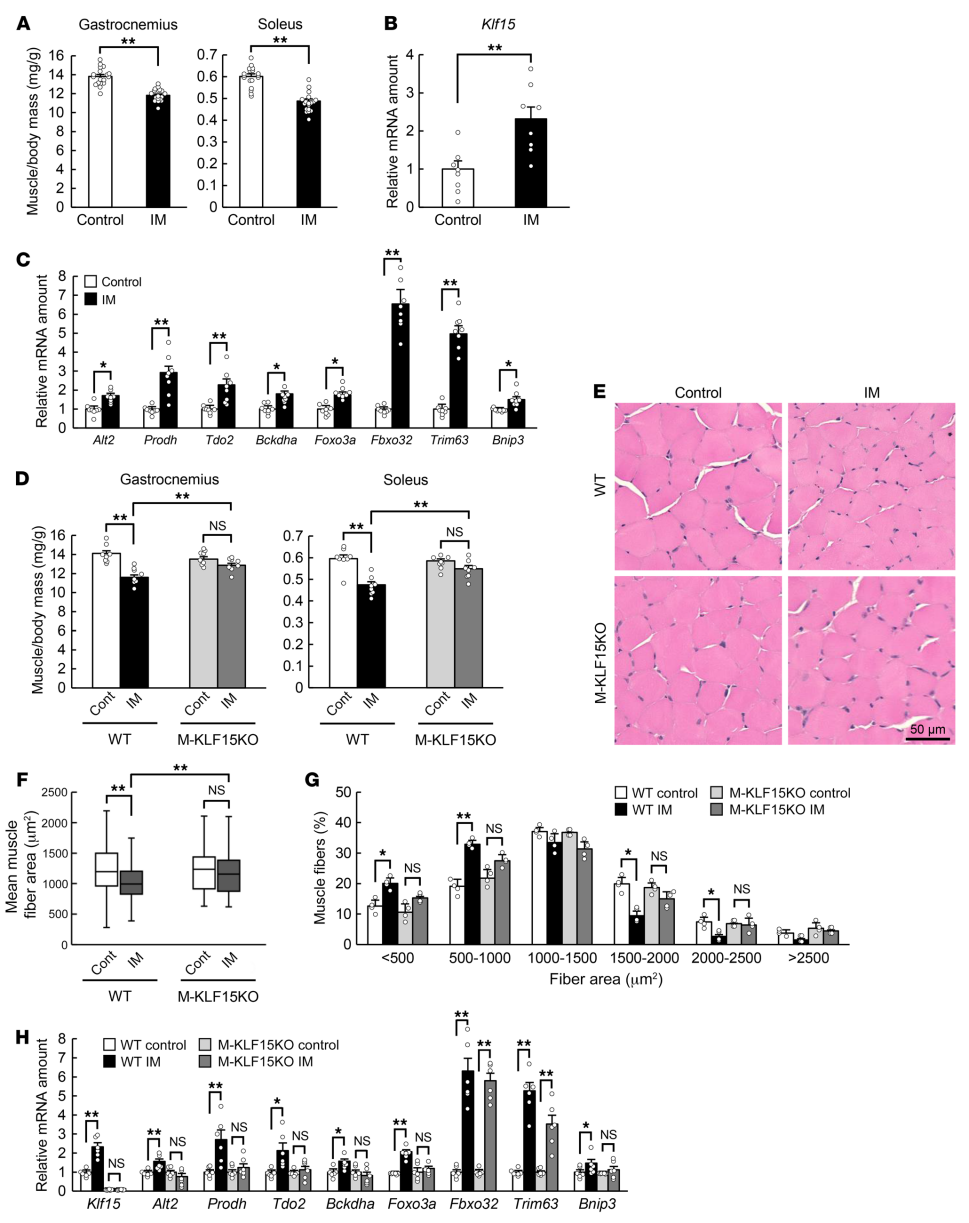
Skeletal muscle atrophy triggered by immobilization is prevented in mice with KLF15 deficiency in skeletal muscle. (**A**) Ratio of gastrocnemius or soleus muscle mass in both hind limbs to body mass for control mice or mice subjected to bilateral hind limb immobilization (IM) with a cast for 3 days (*n* = 18 mice). (**B** and **C**) Quantitative RT-PCR analysis of *Klf15* mRNA (**B**) and of atrophy-related gene expression (**C**) in gastrocnemius of mice as in **A** (*n* = 8 mice). (**D**) Ratio of muscle mass to body mass for WT or M-KLF15KO mice subjected to cast immobilization for 3 days or for corresponding control (Cont) mice (*n* = 8 mice). (**E**–**G**) Hematoxylin-eosin staining (**E**) for determination of muscle fiber area (**F**) and the distribution of muscle fiber area (**G**) in soleus of mice as in **D** (*n* = 8 mice). The area of 800 fibers pooled from 4 mice was measured and averaged for each condition in **F**. Scale bar: 50 μm. (**H**) Quantitative RT-PCR analysis of atrophy-related gene expression in gastrocnemius of mice as in **D** (*n* = 6 mice). Quantitative data are mean ± SEM (**A**–**D**, **G**, and **H**) or medians (**F**). *P < 0.05, **P < 0.01 by unpaired Student’s *t* test (**A**–**C**) or 2-way ANOVA with Bonferroni’s post hoc test (**D** and **F**–**H**). NS, not significant.

Bilateral hind limb immobilization with a cast for 3 days in mice resulted in an approximately 10% to 15% decline in skeletal muscle mass in these limbs compared with those of control mice (Figure 1A). KLF15 was recently shown to contribute to diabetes-induced muscle atrophy (4), and the abundance of *Klf15* mRNA was found to be increased in skeletal muscle of the immobilized mouse limbs (Figure 1B). The expression of genes related to amino acid catabolism (*Alt2*, *Prodh*, *Tdo2*, *Bckdha*), to protein degradation during muscle atrophy (*Foxo3a*, *Fbxo32* [encoding atrogin-1], *Trim63* [encoding MuRF1]), or to autophagy (*Bnip3*) was also increased by immobilization (Figure 1C). Skeletal muscle atrophy triggered by immobilization was prevented in mice lacking KLF15 specifically in skeletal muscle (M-KLF15KO mice), which were generated by crossing mice with a floxed *Klf15* allele (4) with mice that express Cre recombinase under the control of the *Mlc1f* promoter (*Mlc1f-Cre* mice; ref. 5). This effect of KLF15 ablation was apparent at the level of muscle mass (Figure 1D), muscle cross-sectional area determined by computed tomography (CT) (Supplemental Figure 1; supplemental material available online with this article; https://doi.org/10.1172/JCI154611DS1), and muscle fiber area evaluated histologically (Figure 1, E and F). Whereas immobilization increased and decreased the proportions of small and large muscle fibers, respectively, in control mice, no such effects were apparent in M-KLF15KO mice (Figure 1G). The immobilization-induced increase in the expression of muscle atrophy–related genes, with the exception of that for *Fbxo32* and *Trim63*, was also prevented in the mutant mice (Figure 1H). The reduction in skeletal muscle mass (Supplemental Figure 2A) and muscle fiber area (Supplemental Figure 2, B and C) as well as the increase in the expression of muscle atrophy–related genes again with the exception of that for *Fbxo32* and *Trim63* (Supplemental Figure 2D) triggered by denervation-induced immobilization were also abolished in M-KLF15KO mice. These results thus suggested that KLF15 is a key regulator of muscle atrophy induced by immobilization due to physical restraint or nerve damage.

Given that satellite cells of skeletal muscle contribute to the regulation of skeletal muscle mass (6), we investigated the change in the abundance of *Klf15* mRNA in the satellite cell and non–satellite cell fractions, the latter of which is mainly composed of myofiber cells with some commingling of stromal cells and vessel cells. The separation of the 2 fractions was confirmed by the expression of *Pax7*, a marker for satellite cells (Supplemental Figure 3). The upregulation of *Klf15* mRNA during immobilization was observed in the non–satellite cell fraction, but not in the satellite cell fraction (Supplemental Figure 3A). Furthermore, the abundance of *Klf15* mRNA was reduced specifically in the non–satellite cell fraction of M-KLF15KO mice (Supplemental Figure 3B). These results collectively suggest that myofiber cells are primarily involved in the KLF15-mediated processes during skeletal muscle atrophy.

The abundance of *Klf15* mRNA in skeletal muscle is increased by glucocorticoids (7), which also trigger muscle atrophy (8). The plasma concentration of corticosterone was increased in mice in response to cast immobilization (Supplemental Figure 4A), consistent with the notion that immobilization exerts psychophysical stress in mice and therefore stimulates glucocorticoid secretion (9). Surgical removal of the adrenal glands markedly attenuated this increase in the plasma corticosterone level (Supplemental Figure 4A); however, the decline in muscle mass and increase in the expression of atrophy-related genes induced by immobilization were not prevented (Supplemental Figure 4, B and C), indicating that glucocorticoids do not contribute to skeletal muscle atrophy induced by immobilization.

### IL-6 is a downstream effector of KLF15 in immobilization-induced muscle atrophy.

**Figure 2:**
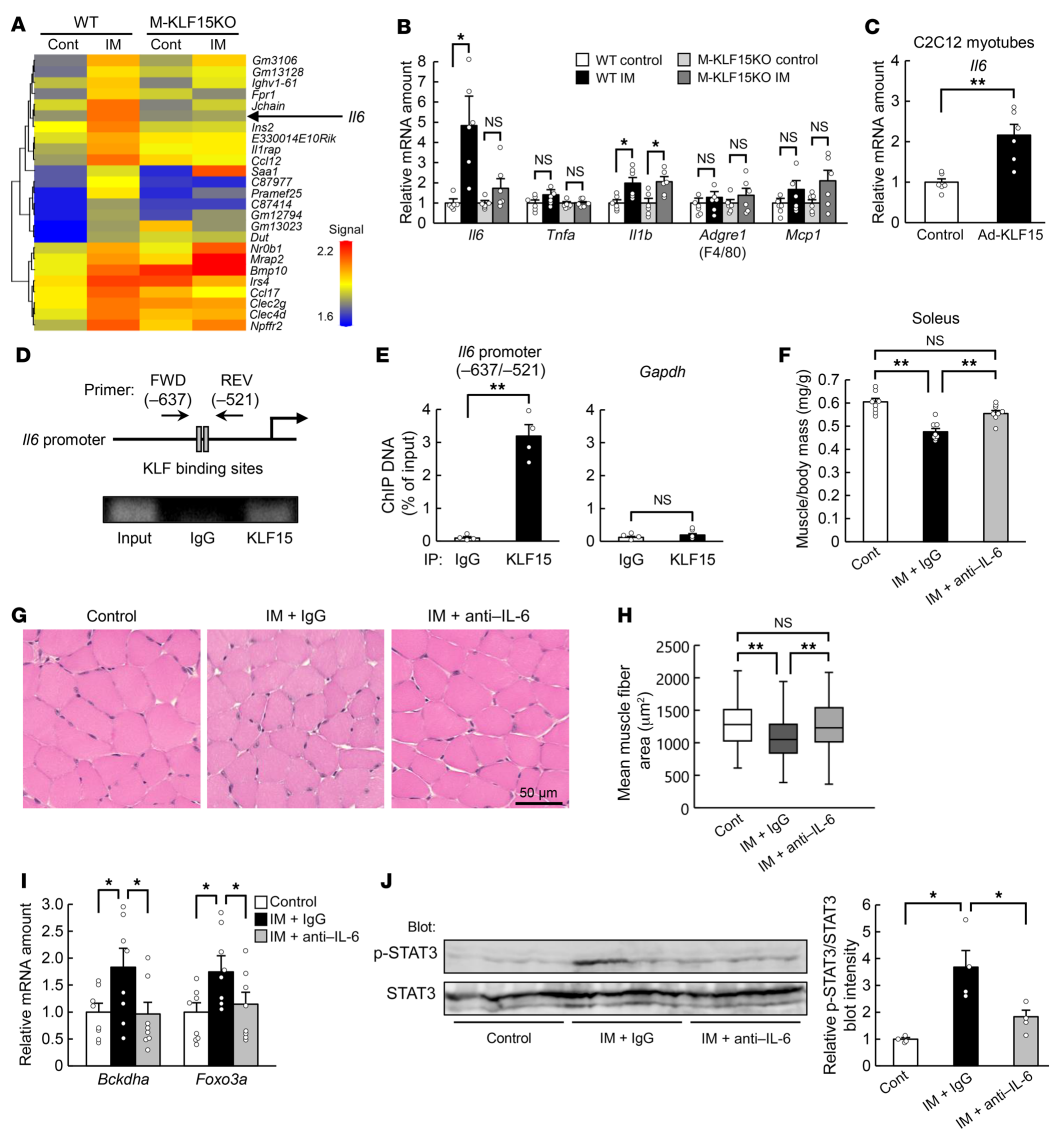
IL-6 is a downstream effector of KLF15 in immobilization-induced muscle atrophy. (**A**) DNA microarray analysis of gene expression in gastrocnemius of WT or M-KLF15KO mice after cast immobilization (IM) of the hind limbs for 3 days. The heatmap shows genes for humoral factors whose expression was upregulated in immobilized WT mice compared with control WT mice. (**B**) Quantitative RT-PCR analysis of inflammation-related gene expression in gastrocnemius of mice as in **A** (*n* = 6 mice). (**C**) Quantitative RT-PCR analysis of *Il6* mRNA (*n* = 6 independent experiments) in C2C12 myotubes infected with an adenovirus encoding LacZ (control) or mouse KLF15 (Ad-KLF15). (**D** and **E**) ChIP assay of KLF15 binding to the mouse *Il6* promoter region in C2C12 myotubes. Immunoprecipitation (IP) was performed with antibodies against KLF15 or with control immunoglobulin G (IgG). A schematic representation of the promoter region indicating the positions of putative KLF binding sites and PCR primers as well as representative gel electrophoresis of PCR products are shown in **D**. Quantitative data for the ChIP analysis of KLF15 binding to the *Il6* promoter region or to *Gapdh* (negative control) are shown in **E** (*n* = 4 independent experiments). (**F**–**J**) Ratio of muscle mass to body mass (*n* = 8 mice) (**F**), histological determination of muscle fiber area in soleus (**G** and **H**), atrophy-related gene expression in gastrocnemius (*n* = 8 mice) (**I**), and immunoblot analysis of total and phosphorylated (p-) forms of STAT3 in gastrocnemius (*n* = 4 mice) (**J**) are shown for control or cast-immobilized mice subjected to intraperitoneal injection of neutralizing antibodies against IL-6 (0.1 mg/mouse) or control IgG at the onset of limb immobilization. Scale bar: 50 μm (**G**). The area of 800 fibers pooled from 4 mice was measured for each condition in **H**. Quantitative data are mean ± SEM (**B**, **C**, **E**, **F**, **I**, and **J**) or medians (**H**). *P < 0.05, **P < 0.01 by unpaired Student’s *t* test (**C** and **E**) or 2-way ANOVA with Bonferroni’s post hoc test (**B**, **F**, and **H**–**J**). NS, not significant.

Skeletal muscle releases humoral factors, known as myokines, that exert various biological actions (10). To search for myokines that might be related to immobilization-induced muscle atrophy, we performed a comprehensive transcriptome analysis. Microarray analysis revealed that the expression of many genes for humoral factors was upregulated in skeletal muscle of WT mice in response to immobilization (Figure 2A). Among these genes, the immobilization-induced expression of *Il6* was prevented in M-KLF15KO mice (Figure 2A), suggesting that the expression of *Il6* is under the control of KLF15. KLF15-dependent regulation of *Il6* expression in response to immobilization was confirmed by quantitative reverse transcription PCR (RT-PCR) analysis (Figure 2B). We also examined the effect of immobilization of the expression of other genes for inflammatory proteins, but none of those tested showed a pattern of change similar to that apparent for *Il6* (Figure 2B). Overexpression of KLF15 in C2C12 myotubes increased the amount of *Il6* mRNA (Figure 2C), and chromatin immunoprecipitation (ChIP) analysis revealed that KLF15 binds to the promoter region of the *Il6* gene (Figure 2, D and E).

Although the plasma concentration of IL-6 was not significantly increased in response to immobilization (Supplemental Figure 5A), administration of neutralizing antibodies against IL-6 prevented immobilization-induced skeletal muscle atrophy (Figure 2, F–H) as well as upregulation of atrophy-related genes (Figure 2I). The antibodies against IL-6 had no effect on the mass or the abundance of atrophy-related genes in skeletal muscle of control mice (Supplemental Figure 5, B and C) or immobilized M-KLF15KO mice (Supplemental Figure 5, D and E), consistent with the notion that IL-6 is a downstream effector of KLF15-mediated muscle atrophy during immobilization.

Immobilization also induced the phosphorylation of signal transducer and activator of transcription 3 (STAT3), a key player in IL-6 signaling, in skeletal muscle, and this effect was prevented by neutralizing antibodies against IL-6 (Figure 2J) as well as in M-KLF15KO mice (Supplemental Figure 6A). Furthermore, immobilization-induced muscle atrophy (Supplemental Figure 6, B–D) as well as expression of muscle atrophy–related genes (Supplemental Figure 6E) and STAT3 phosphorylation (Supplemental Figure 6F) in skeletal muscle were all abolished in *Il6*-KO mice. Together, these results suggested that IL-6 is induced directly by KLF15 during immobilization and contributes to the development of muscle atrophy in an autocrine or paracrine manner.

### A reduction in [Ca^2+^]_i_ to below the basal level triggers muscle atrophy.

**Figure 3:**
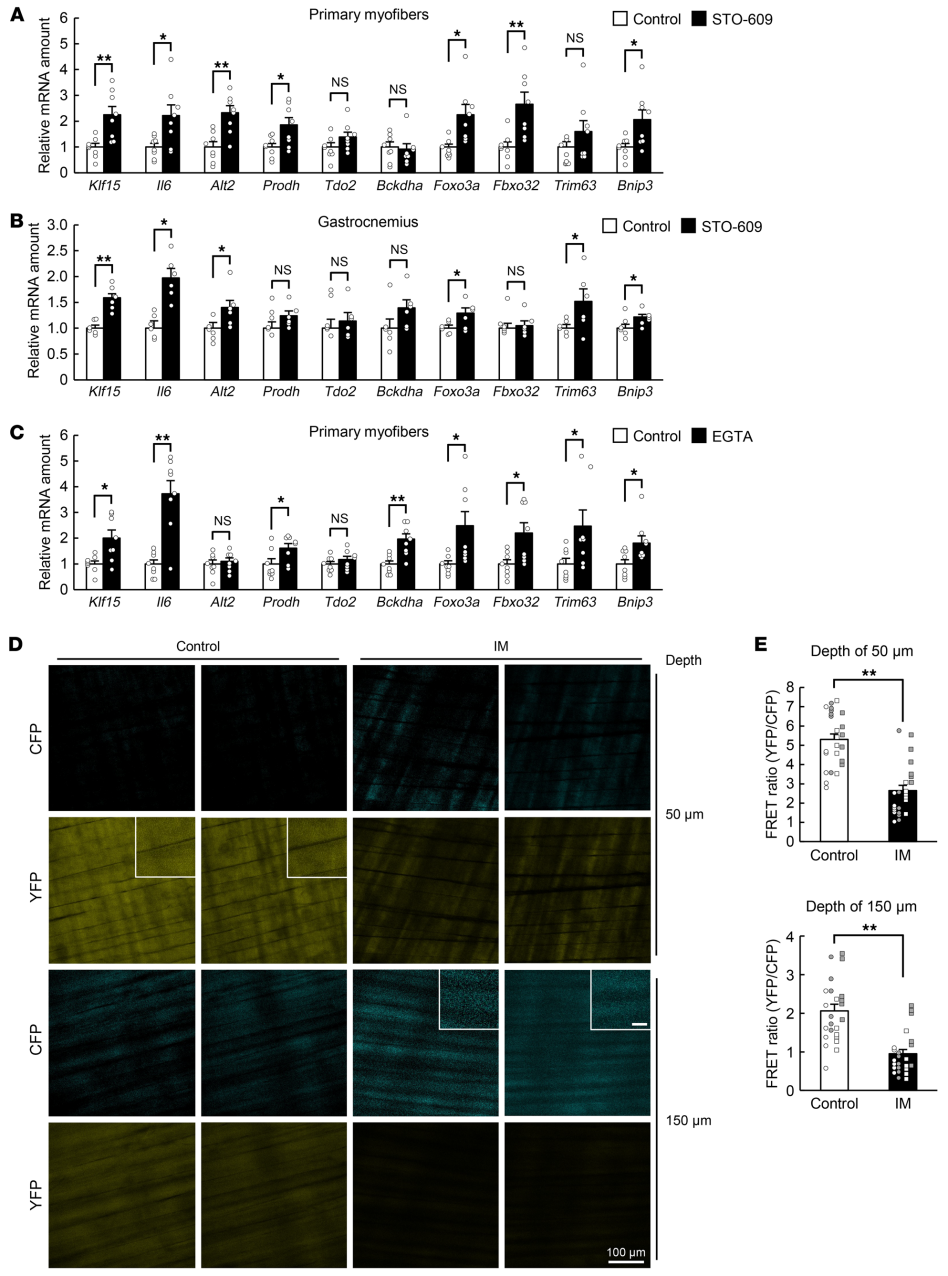
A decrease in [Ca^2+^]_i_ to below the basal level is associated with muscle atrophy. (**A** and **C**) Quantitative RT-PCR analysis of the expression of atrophy-related genes, including *Klf15* and *Il6*, in mouse primary myofibers exposed to 2 μM STO-609 or vehicle (control) for 24 hours (*n* = 8 independent experiments) (**A**) or to 0.1 mM EGTA or vehicle (control) for 3 hours (*n* = 8 independent experiments) (**C**). (**B**) Quantitative RT-PCR analysis of atrophy-related gene expression in gastrocnemius of WT mice at 6 hours after intraperitoneal injection of STO-609 (10.4 mg/kg) or vehicle (control) (*n* = 6 mice). (**D** and **E**) Intravital Ca^2+^ imaging of M-YC3.60–Tg mice. Representative 2-photon images of CFP and YFP fluorescence at a depth of 50 or 150 μm from the fascia of the tibialis anterior muscle subjected (or not, control) to immobilization (IM) for 24 hours are shown in **D**. Scale bars: 100 μm (main panels) and 20 μm (insets). Quantitation of the FRET ratio in areas of 6 fibers for each of 4 mice is shown in **E**, with white or gray circles or squares indicating the values obtained from individual animals. All quantitative data are mean ± SEM. *P < 0.05, **P < 0.01 by unpaired Student’s *t* test. NS, not significant.

Given that Ca^2+^ signaling has been implicated in the regulation of skeletal muscle mass (11), we examined the possible role of such signaling in muscle atrophy. Treatment of mouse primary myofibers (Figure 3A) or C2C12 myotubes (Supplemental Figure 7A) with a pharmacological inhibitor of Ca^2+^/calmodulin-dependent protein kinase kinase (CaMKK), a key molecule in Ca^2+^ signaling, resulted in upregulation of the expression of genes related to muscle atrophy, including *Klf15* and *Il6*. Administration of the same inhibitor (STO-609) to mice also mimicked the effect of limb immobilization on atrophy-related gene expression in skeletal muscle (Figure 3B). In addition, treatment of mouse primary myofibers (Figure 3C) or C2C12 myotubes (Supplemental Figure 7B) with the Ca^2+^ chelator EGTA increased the expression of atrophy-related genes, further implicating Ca^2+^ signaling in muscle atrophy.

Given that the CaMKK inhibitor and EGTA each upregulated the expression of *Klf15* in mouse primary myofibers in the absence of any specific stimulus, we hypothesized that a reduction in [Ca^2+^]_i_ from the basal level might induce the expression of *Klf15* and skeletal muscle atrophy. To test this hypothesis, we established a bioimaging technique to measure [Ca^2+^]_i_ in skeletal muscle under static conditions in living animals. Yellow Cameleon 3.60 (YC3.60) is a fusion protein composed of cyan fluorescent protein (CFP), calmodulin (CaM), the M13 peptide of myosin light chain kinase, and yellow fluorescent protein (YFP) (12), and excitation of CFP in the presence of Ca^2+^ results in fluorescence resonance energy transfer (FRET) to YFP. Mice harboring a construct encoding floxed *YC3.60* express the fusion protein under the control of the CAG promoter in cells that also express Cre recombinase (Supplemental Figure 8A and ref. 13). We therefore crossed these mice with *Mlc1f-Cre* mice to generate animals in which YC3.60 is specifically expressed in skeletal muscle (M-YC3.60–Tg mice). We subjected the M-YC3.60–Tg mice to unilateral hind limb immobilization with a cast for 24 hours and then measured the fluorescence of CFP and YFP for calculation of the FRET ratio in the immobilized and contralateral control limbs (Supplemental Figure 8, B–D). The mice were anesthetized for this analysis in order to render skeletal muscle flaccid and allow measurement of [Ca^2+^]_i_ in the static condition. Two-photon microscopy detected fluorescence of CFP and YFP in the tibialis anterior muscle of the control limb at depths of 50 and 150 μm, indicating that this imaging technique is sufficiently sensitive for measurement of basal [Ca^2+^]_i_. The fluorescence of CFP was increased and that of YFP decreased, resulting in a decrease in the FRET ratio, in the immobilized limb compared with the control limb (Figure 3, D and E), indicating that immobilization elicits a lowering of [Ca^2+^]_i_ in skeletal muscle under the static condition.

### Piezo1 contributes to maintenance of basal [Ca^2+^]_i_ and to immobilization-induced muscle atrophy.

**Figure 4:**
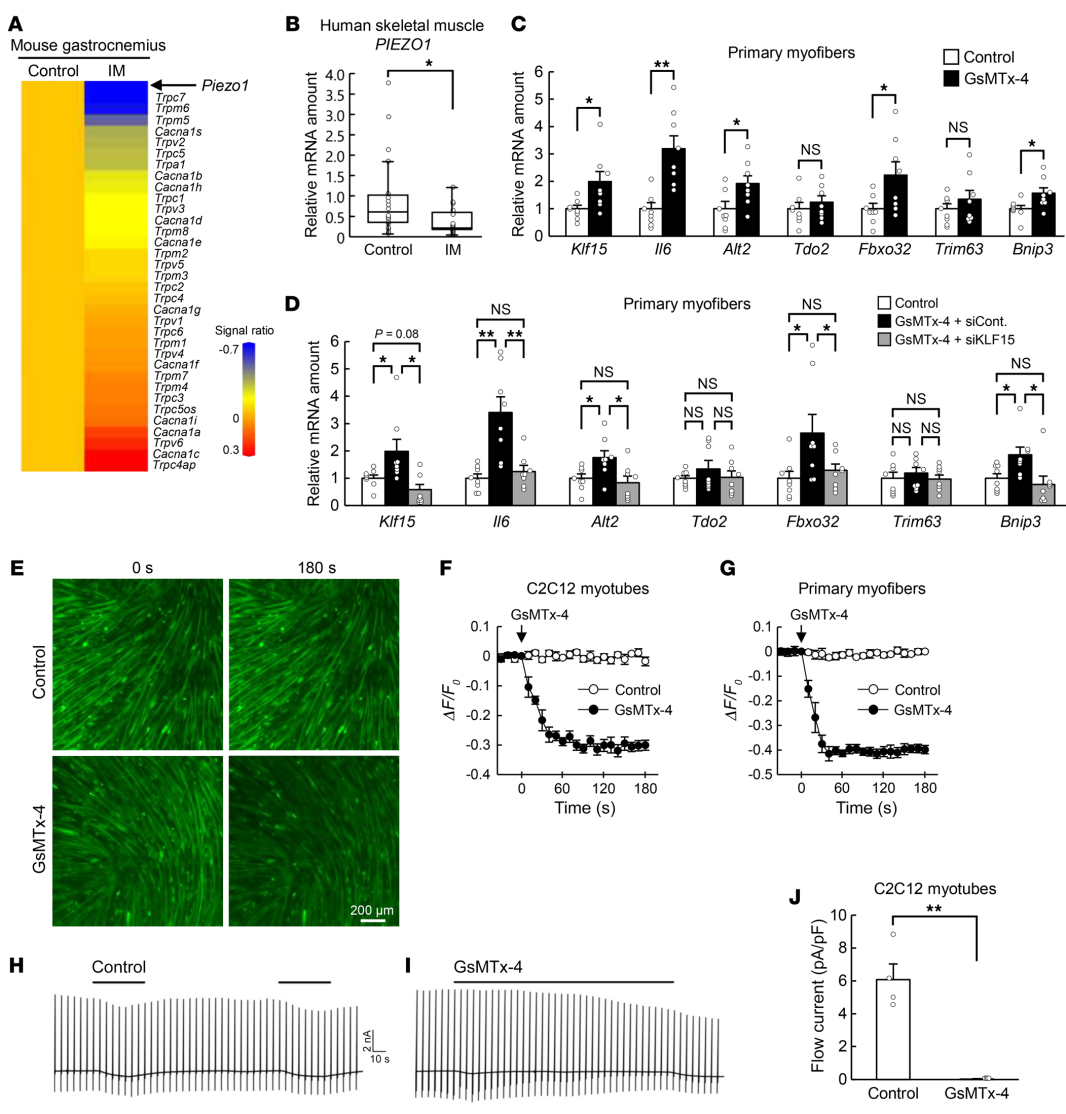
Piezo1 regulates muscle atrophy–related gene expression in a KLF15-dependent manner. (**A**) Heatmap for DNA microarray analysis of downregulated (top) and upregulated (bottom) genes for Ca^2+^ channels of the cell membrane in gastrocnemius of mice subjected to cast immobilization (IM) for 3 days compared with control mice. (**B**) Quantitative RT-PCR analysis of *PIEZO1* mRNA in skeletal muscle of control (*n* = 18) or immobilized (*n* = 15) human participants. (**C**) Quantitative RT-PCR analysis of the expression of atrophy-related genes, including *Klf15* and *Il6*, in mouse primary myofibers exposed to 50 μM GsMTx-4 or vehicle (control) for 6 hours (*n* = 8 independent experiments). (**D**) Quantitative RT-PCR analysis of the expression of atrophy-related genes, including *Klf15* and *Il6*, in mouse primary myofibers exposed to vehicle (control) or 50 μM GsMTx-4 as well as those transfected with control (siCont) or KLF15 (siKLF15) siRNAs for 6 hours (*n* = 8 independent experiments). (**E**–**G**) Fluorescence microscopic images of C2C12 myotubes loaded with Fluo-8 were obtained before and after exposure to 10 μM GsMTx-4 or vehicle (control) for 180 seconds (**E**). Scale bar: 200 μm. The time course of fluorescence intensity was also measured in C2C12 myotubes (*n* = 4 independent experiments) (**F**) or mouse primary myofibers (*n* = 4 independent experiments) (**G**). (**H**–**J**) Representative whole-cell patch-clamp traces show that application of flow (black bars) induced inward currents in C2C12 myotubes (control) (**H**), and that such currents were inhibited by GsMTx-4 (5 μM) (**I**). Ramp pulses (from –100 to +100 mV for 500 ms) were applied at 5-second intervals. The holding potential was –60 mV. The amplitude (as current density) of flow-induced currents in both control and GsMTx-4 conditions was quantified (*n* = 4 independent experiments) (**J**). Quantitative data are medians (**B**) or mean ± SEM (**C**, **D**, **F**, **G**, and **J**). *P < 0.05, **P < 0.01 by Mann-Whitney *U* test (**B**), unpaired Student’s *t* test (**C** and **J**), or 2-way ANOVA with Bonferroni’s post hoc test (**D**). NS, not significant.

Given that EGTA does not enter cells and thus chelates only extracellular Ca^2+^, we concluded that Ca^2+^ influx into cells is a key determinant of [Ca^2+^]_i_ in skeletal muscle under the static condition. We therefore examined the effects of cast immobilization on the expression of genes encoding membrane Ca^2+^ channels in gastrocnemius with the use of microarray analysis (Figure 4A). Among such genes whose expression was downregulated by immobilization, *Piezo1* showed the greatest such change (Figure 4A). Downregulation of *Piezo1* expression in response to cast immobilization was confirmed by quantitative RT-PCR analysis (Supplemental Figure 9A), which also revealed a similar effect of denervation-induced limb immobilization (Supplemental Figure 9B). Furthermore, downregulation of *Piezo1* was observed in the non–satellite cell fraction, but not in the satellite cell fraction during immobilization-induced muscle atrophy (Supplemental Figure 9C).

We then analyzed the expression of *PIEZO1* in human skeletal muscle. Skeletal muscle biopsy samples were obtained under general anesthesia from individuals who had undergone cast fixation for a bone fracture, a procedure that rapidly results in pronounced muscle atrophy, as well as from control participants who had recovered from muscle atrophy (Supplemental Table 1). The abundance of *PIEZO1* mRNA in skeletal muscle was smaller for the patients who had undergone cast fixation than for the controls (Figure 4B), suggestive of a pathophysiological role for Piezo1 in immobilization-induced muscle atrophy in humans.

Treatment of mouse primary myofibers (Figure 4C) or C2C12 myotubes (Supplemental Figure 9D) with GsMTx-4, a pharmacological inhibitor of Piezo1, increased the expression of atrophy-related genes, including *Klf15* and *Il6*. Nifedipine and tranilast — inhibitors of L-type voltage-dependent Ca^2+^ channels and TRPV2 (transient receptor potential cation channel, subfamily V, member 2), respectively — did not manifest such effects (Supplemental Figure 9, E and F). The upregulation of gene expression in mouse primary myofibers (Figure 4D) or C2C12 myotubes (Supplemental Figure 9G) by GsMTx-4 was attenuated by small interfering RNA–mediated (siRNA-mediated) knockdown of KLF15, indicating that the effect of Piezo1 inhibition on atrophy-related gene expression is mediated by KLF15.

We next investigated whether Piezo1 contributes to the regulation of basal [Ca^2+^]_i_ in skeletal muscle. We first utilized the Ca^2+^ indicator Fluo-8, which has been applied to monitor [Ca^2+^]_i_ in cultured muscle cells (14). Intracellular Fluo-8 fluorescence decreased rapidly on exposure of C2C12 myotubes or mouse primary myofibers to GsMTx-4 (Figure 4, E–G), indicating that Piezo1 plays an important role in the maintenance of basal [Ca^2+^]_i_.

Piezo1 is activated by mechanical stimuli (15, 16). Whole-cell patch-clamp analysis revealed that flow application as a mechanical stimulus induced a substantial inward current in C2C12 myotubes (Figure 4, H and J). To characterize the current-voltage relation, we applied ramped pulses from –100 to +100 mV at 5-second intervals. The flow-induced current was markedly inhibited in the presence of GsMTx-4 (Figure 4, I and J), indicating that Piezo1 mediates cellular current in C2C12 myotubes in response to mechanical stimulation.

**Figure 5:**
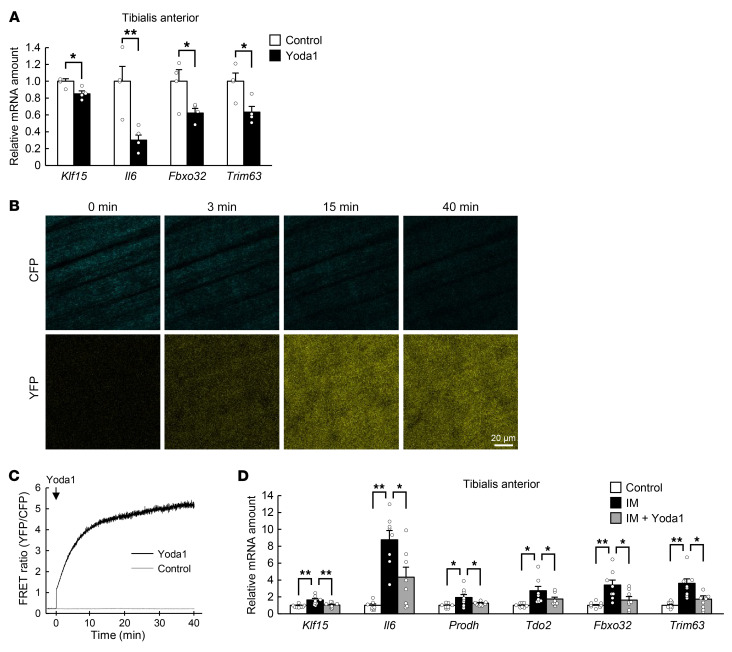
Effect of a Piezo1 channel activator on immobilization-induced muscle atrophy. (**A**) Quantitative RT-PCR analysis of the expression of atrophy-related genes, including *Klf15* and *Il6*, in the tibialis anterior muscle of WT mice at 6 hours after intramuscular injection of Yoda1 (0.2 mg/kg) or vehicle (control) (*n* = 4 mice). (**B** and **C**) Intravital Ca^2+^ imaging of M-YC3.60–Tg mice. Representative 2-photon images of CFP and YFP fluorescence at the indicated times after intramuscular injection of Yoda1 at 0.2 mg/kg (**B**) as well as the time course of the FRET ratio (*n* = 4 mice) (**C**) are shown for the tibialis anterior of M-YC3.60–Tg mice. Scale bar: 20 μm. (**D**) Quantitative RT-PCR analysis of the expression of atrophy-related genes, including *Klf15* and *Il6*, in tibialis anterior of control mice or mice subjected to cast immobilization (IM) for 3 days with or without intramuscular injection of Yoda1 (0.2 mg/kg) at the onset of immobilization (*n* = 8 mice). All quantitative data are mean ± SEM. *P < 0.05, **P < 0.01 by unpaired Student’s *t* test (**A**) or 2-way ANOVA with Bonferroni’s post hoc test (**D**).

Treatment of C2C12 myotubes with Yoda1, an activator of Piezo1 (17), resulted in the downregulation of atrophy-related gene expression (Supplemental Figure 9H). Intramuscular injection of Yoda1 also induced downregulation of the expression of atrophy-related genes in skeletal muscle (Figure 5A). Such injection of Yoda1 also increased [Ca^2+^]_i_ in muscle cells of M-YC3.60–Tg mice (Figure 5, B and C). Furthermore, intramuscular administration of Yoda1 suppressed the immobilization-induced increase in the expression of atrophy-related genes (Figure 5D).

We next examined the effects of acute deficiency of Piezo1 in living animals. We crossed *Piezo1*-floxed mice with *HSA-Cre-ERT2* mice (18) so as to be able to induce downregulation of *Piezo1* expression in skeletal muscle of the resultant iM-Piezo1KO mice by administration of tamoxifen (Supplemental Figure 10, A–C). Administration of tamoxifen to iM-Piezo1KO mice resulted in a decrease in the abundance of *Piezo1* mRNA in skeletal muscle of only approximately 30% to 60% (Supplemental Figure 10D), however, possibly as a result of incomplete gene recombination. The decrease in the abundance of *Piezo1* mRNA was apparent only in the non–satellite cell fraction of skeletal muscle (Supplemental Figure 10E).

**Figure 6:**
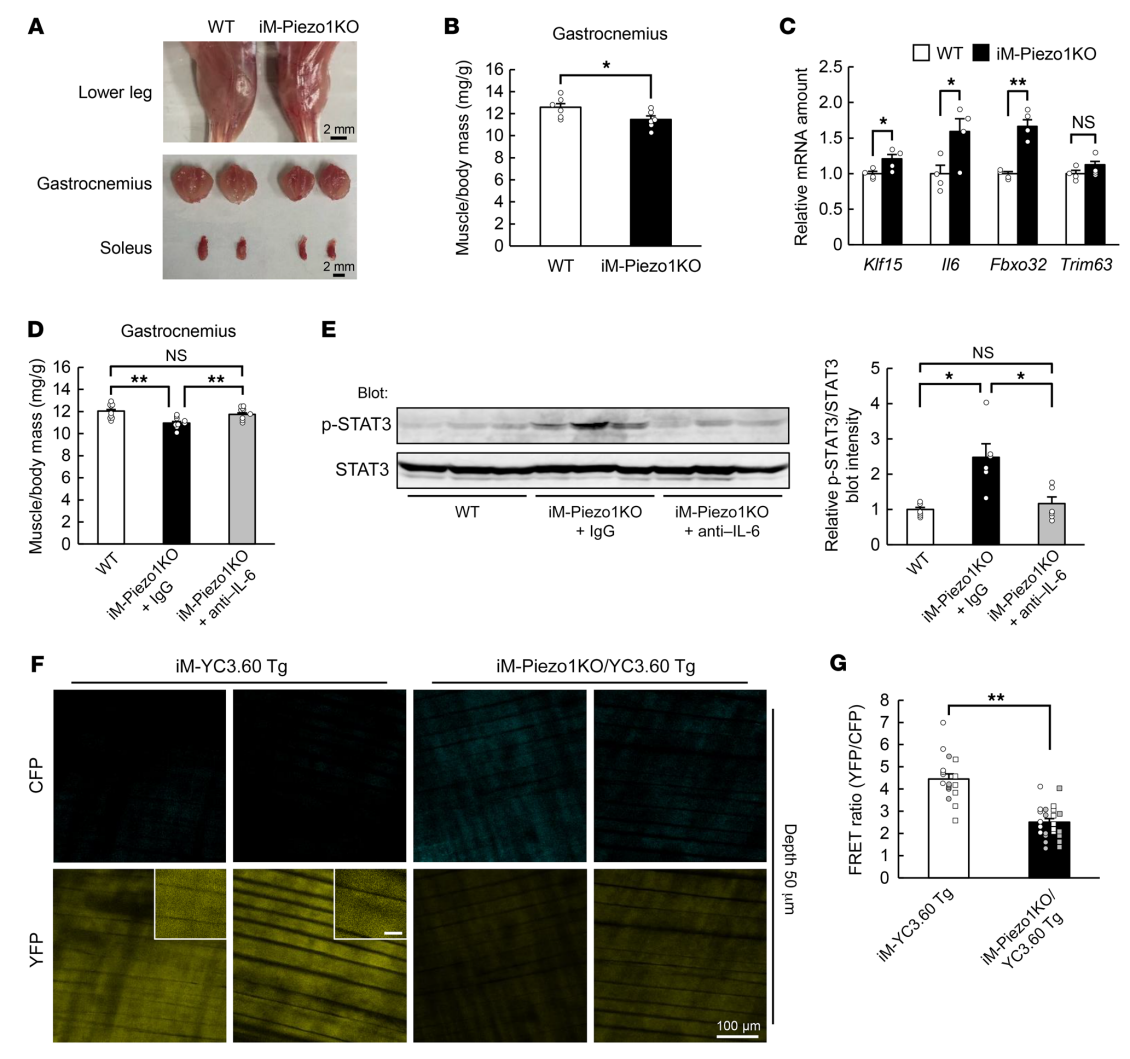
The phenotype of tamoxifen-inducible skeletal muscle–specific Piezo1 KO (iM-Piezo1KO) mice. (**A**–**C**) Representative images of the lower hind limb, gastrocnemius, and soleus (**A**), the ratio of gastrocnemius muscle mass to body mass (*n* = 6 mice) (**B**), and quantitative RT-PCR analysis of the expression of atrophy-related genes, including *Klf15* and *Il6*, in gastrocnemius (*n* = 4 mice) (**C**) for WT or tamoxifen-treated iM-Piezo1KO mice. Scale bars: 2 mm. (**D** and **E**) Ratio of muscle mass to body mass (*n* = 9 mice) (**D**), and immunoblot analysis of total and phosphorylated (p-) forms of STAT3 in gastrocnemius (*n* = 6 mice) (**E**) are shown for WT or iM-Piezo1KO mice subjected to intraperitoneal injection of neutralizing antibodies against IL-6 (0.1 mg/mouse) or control IgG at the onset of tamoxifen treatment. (**F** and **G**) Intravital Ca^2+^ imaging of iM-Piezo1KO/YC3.60–Tg mice. Representative 2-photon images of CFP and YFP fluorescence at a depth of 50 μm from the fascia of the tibialis anterior muscle for tamoxifen-treated iM-YC3.60–Tg mice or iM-Piezo1KO/YC3.60–Tg mice are shown in **F**. Scale bars: 100 μm (main panels) and 20 μm (insets). Quantitation of the FRET ratio in areas of 6 fibers for each of 3 (iM-YC3.60 Tg) or 4 (iM-Piezo1KO/YC3.60 Tg) hind limbs is shown in **G**, with white or gray circles or squares indicating the values obtained from individual hind limbs. All quantitative data are mean ± SEM. *P < 0.05, **P < 0.01 by unpaired Student’s *t* test (**B**, **C**, and **G**) or 2-way ANOVA with Bonferroni’s post hoc test (**D** and **E**). NS, not significant.

Whereas the body mass of mice with acute *Piezo1* ablation in skeletal muscle did not differ from that of control mice (Supplemental Figure 10F), skeletal muscle mass (Figure 6, A and B, and Supplemental Figure 11A) and muscle fiber area (Supplemental Figure 11, B and C) were smaller in the mutant mice than in control mice. The expression of atrophy-related genes, including *Klf15* and *Il6*, was also upregulated in skeletal muscle in response to acute *Piezo1* ablation (Figure 6C). These results thus indicated that the downregulation of *Piezo1* expression to an extent similar to that induced by immobilization — about 30% in mice (Supplemental Figure 9, A and B) and approximately 60% in humans (Figure 4B) — is sufficient to trigger skeletal muscle atrophy. Furthermore, skeletal muscle atrophy (Figure 6D), upregulation of atrophy-related genes (Supplemental Figure 11D), and phosphorylation of STAT3 in skeletal muscle (Figure 6E) induced by the downregulation of *Piezo1* were prevented by the administration of neutralizing antibodies against IL-6, confirming that IL-6 is a downstream effector of Piezo1 in skeletal muscle atrophy.

We next crossed *YC3.60*-floxed mice, *Piezo1*-floxed mice, and *HSA-Cre-ERT2* mice to generate mice in which YC3.60 protein and Piezo1 are concomitantly induced and suppressed, respectively, in skeletal muscle by administration of tamoxifen. We administered tamoxifen to the resultant mice (iM-Piezo1KO/YC3.60–Tg mice) and analyzed [Ca^2+^]_i_ under anesthesia. Two-photon microscopy analysis revealed that the fluorescence of CFP was increased and that of YFP was decreased, resulting in a decrease in the FRET ratio, in skeletal muscle of the mutant mice (Figure 6, F and G, and Supplemental Figure 11, E and F), indicating that Piezo1 plays a key role in the maintenance of [Ca^2+^]_i_ in skeletal muscle of living animals under the static condition.

### Changes in gene expression in skeletal muscle of patients with immobility-induced muscle atrophy.

**Figure 7:**
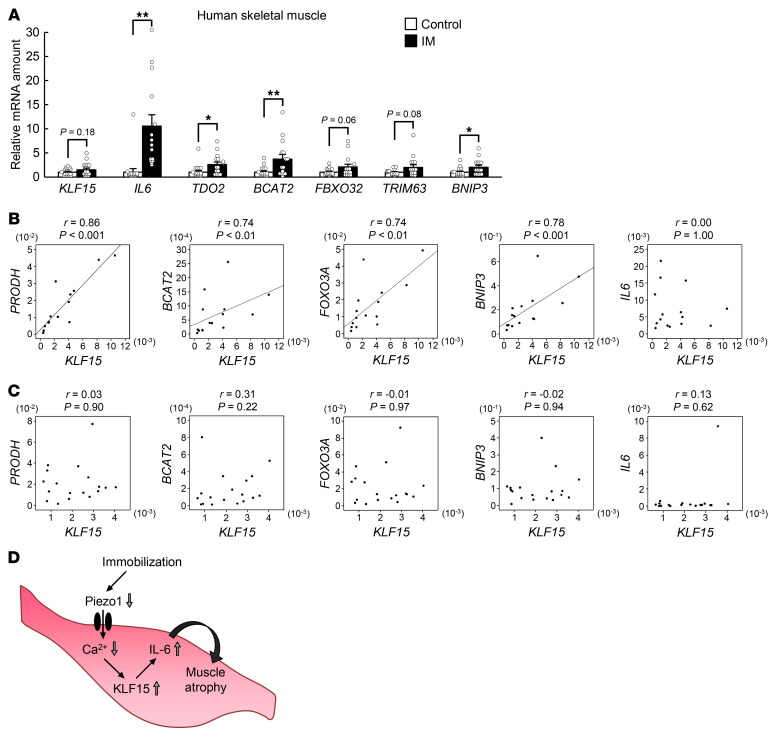
Role of KLF15 in regulation of muscle atrophy–related gene expression in humans. (**A**) Quantitative RT-PCR analysis of atrophy-related genes, including *KLF15* and *IL6*, in skeletal muscle of control (*n* = 18) or immobilized (IM, *n* = 15) human participants. Data are mean ± SEM. *P < 0.05, **P < 0.01 by unpaired Student’s *t* test. (**B** and **C**) Spearman’s rank correlation analysis for the expression of *KLF15* and that of other muscle atrophy–related genes in skeletal muscle of immobilized patients (*n* = 15) (**B**) or control participants (*n* = 18) (**C**). (**D**) Proposed role for a Piezo1/KLF15/IL-6 axis in immobilization-induced muscle atrophy.

Finally, we investigated gene expression in skeletal muscle of patients who had undergone cast immobilization for bone fractures. In addition to the downregulation of *PIEZO1* expression (Figure 4B), the expression of *IL6* as well as that of other genes related to muscle atrophy, including *TDO2*, *BCAT2*, and *BNIP3*, was upregulated in skeletal muscle of these individuals (Figure 7A). The expression of *KLF15*, *FBXO32*, and *TRIM63* also tended to be increased, although these changes did not achieve statistical significance. The expression of *KLF15* and that of the atrophy-related genes *PRODH*, *BCAT2*, *FOXO3A*, and *BNIP3* was significantly correlated in these patients (Figure 7B). Such a correlation was not apparent in control subjects (Figure 7C). No significant correlation was apparent between the expression of *KLF15* and *IL6* in patients with or without atrophy (Figure 7, B and C), possibly due to the considerable variation of the expression of *IL6* in the samples. These results thus implicated the Piezo1/KLF15/IL-6 axis in immobilization-induced muscle atrophy in humans.

## Discussion

We have here shown that immobilization reduces the [Ca^2+^]_i_ of skeletal muscle cells, likely as a result, at least in part, of downregulation of Piezo1. This reduction in [Ca^2+^]_i_, in turn, promotes atrophy of skeletal muscle in a manner dependent on KLF15 and IL-6 (Figure 7D). Moreover, the contribution of the Piezo1/KLF15/IL-6 axis to immobilization-induced muscle atrophy identified in mice was validated with the use of biopsy samples of patients with cast fixation–induced skeletal muscle atrophy.

Ca^2+^-dependent intracellular processes are generally triggered by an acute and robust increase in [Ca^2+^]_i_ from the tens of nanomolar range to the micromolar range. Given that the Ca^2+^ chelator EGTA, as well as inhibitors of CaMKK and Piezo1, upregulated the expression of atrophy-related genes in nonstimulated cultured muscle cells, we hypothesized that a decrease in [Ca^2+^]_i_ to a level below the basal concentration is a trigger for the development of immobilization-induced muscle atrophy. A newly developed Ca^2+^ imaging technique indeed revealed that the [Ca^2+^]_i_ in skeletal muscle was lower in immobilized or Piezo1-deficient mouse limbs than in control limbs even under anesthesia, which renders skeletal muscle flaccid. Whereas previous studies examined the [Ca^2+^]_i_ in isolated skeletal muscle (19, 20), the present study analyzed the dynamics of [Ca^2+^]_i_ in skeletal muscle in living animals. Evidence has suggested that not only an increase but also a decrease in [Ca^2+^]_i_ from the basal level can modulate cellular functions (21, 22). Our results provide a link between a decrease in [Ca^2+^]_i_ and a defined biological event, however.

Piezo1 is activated by mechanical stimuli (15, 16), and we indeed found that it is responsible for an inward current induced by mechanical stimulation in C2C12 myotubes. However, our finding that GsMTx-4 lowered [Ca^2+^]_i_ in C2C12 myotubes or mouse primary myofibers indicates that Piezo1 is active, at least to some extent, even in the absence of a specific mechanical stimulus and contributes to maintenance of the basal [Ca^2+^]_i_ in muscle cells. Whereas Piezo1 has previously been shown to regulate physiological phenomena in cardiac (23) or smooth muscle (24) in response to mechanical stimuli, we have here uncovered a function of Piezo1 in skeletal muscle under the static condition. Given that voluntary movement in mice likely results in mechanical stimulation of skeletal muscle, the immobilization-induced decrease in [Ca^2+^]_i_ in muscle cells is possibly attributable both to downregulation of Piezo1 and to attenuation of mechanical stimuli. The relative contributions of these 2 factors remain to be elucidated. The mechanism by which Piezo1 is downregulated in response to immobilization also remains unknown. Given that mechanical stimuli appear to contribute not only to the activity but also to the abundance of Piezo1 (25–27), the lack of mechanical stimuli during immobilization may be responsible for its downregulation.

We recently showed that the abundance of KLF15 in skeletal muscle is increased in response to hyperglycemia as a result of suppression of its ubiquitination by the E3 ubiquitin ligase WWP1, which in turn contributes to the development of skeletal muscle atrophy in diabetes mellitus (4). KLF15 is also implicated in glucocorticoid-induced muscle atrophy (7, 28). These findings suggest that this transcription factor plays an important role in the development of muscle atrophy associated with various conditions, whereas upstream regulatory mechanisms appear to differ among such conditions. IL-6 is implicated in muscle atrophy associated with cancer-induced cachexia (29), chronic inflammatory diseases (30), and immobilization (31). Our results now suggest that IL-6 is induced in skeletal muscle in a cell-autonomous manner via a Piezo1/KLF15 pathway. Given that IL-6 secreted from fibro-adipogenic progenitor cells in skeletal muscle has previously been found to contribute to denervation-induced muscle atrophy (32), which cell types are responsible for IL-6 secretion during muscle immobilization remains to be clarified.

Although the expression of *PIEZO1* and that of certain atrophy-related genes, including *IL6* and *BNIP3*, was significantly decreased and increased, respectively, in skeletal muscle of patients who had undergone cast fixation, observed increases in the expression of *KLF15*, *FBXO32*, and *TRIM63* were not significant. Such differences in the effects of immobilization on gene expression might be attributable to heterogeneity of the study participants. Although it is well established that cast fixation rapidly induces muscle atrophy in humans, information on the extent of muscle mass decline was not available for the current study participants. Analysis of patients with a more homogeneous clinical background and with available information on the extent of muscle mass decline is warranted to further confirm our present findings.

In summary, we have here uncovered a phenomenon in cellular Ca^2+^ signaling: a decrease in [Ca^2+^]_i_ from the basal level that appears to trigger the development of muscle atrophy in response to immobilization. It will be of interest to determine whether such signaling might also contribute to other physiological or pathological conditions. Given that a decline in muscle mass plays a role in the development or exacerbation of many health problems, the pathway uncovered here may serve as a therapeutic target for muscle mass decline and its associated pathological conditions.

## Methods

### Animals.

*Klf15*-floxed mice (4), *Il6*-KO mice (33), *YC3.60*-floxed mice (13), *Mlc1f-Cre* mice (5), and *HSA-Cre-ERT2* mice (18) were described previously. *Piezo1*-floxed mice were obtained from the Mutant Mouse Resource & Research Center (University of California, Davis). All experiments were performed with male mice at 10 weeks of age unless indicated otherwise. Given that unilateral immobilization of a hind limb results in compensatory hypertrophy of skeletal muscle in the contralateral limb (34), we selected bilateral hind limb immobilization with hind limbs of nonimmobilized animals as control unless indicated otherwise. Mice were anesthetized by intraperitoneal injection of a mixture of 3 anesthetic agents (0.3 mg/kg medetomidine, 4.0 mg/kg midazolam, and 5.0 mg/kg butorphanol) and then subjected to bilateral cast immobilization of hind limbs with the use of plastic tubes and adhesive tape, or to bilateral hind limb denervation by transection of the sciatic nerve. The immobilized mice were able to move freely in the cage and to obtain food and water using their fore limbs. Animals were analyzed 3 days (unless indicated otherwise) after cast immobilization or 2 days after denervation. Cast-immobilized or control mice were injected intraperitoneally with neutralizing antibodies against IL-6 (MP5-20F3, R&D Systems) at 0.1 mg/mouse or with STO-609 (Cayman Chemical) at 10.4 mg/kg, or they were injected intramuscularly with Yoda1 (Cayman Chemical) at 0.2 mg/kg. To examine the effects of immobilization on [Ca^2+^]_i_, mice were subjected to unilateral cast immobilization of a hind limb for 24 hours because the change in [Ca^2+^]_i_ should precede muscle atrophy if it is a cause of muscle atrophy. We utilized the contralateral limb as the control in this experiment, given that unilateral immobilization does not cause compensatory hypertrophy in the contralateral limb within 24 hours (34). Adrenalectomy or sham surgery was performed under anesthesia as described above. The separation of the satellite cell and the non–satellite cell muscle tissue fractions was performed with the use of a Satellite Cell Isolation Kit (Miltenyi Biotec) and an autoMACS Pro Separator (Miltenyi Biotec). Plasma corticosterone and IL-6 concentrations were measured with an enzyme immunoassay kit (Yanaihara Institute) and an enzyme-linked immunosorbent assay kit (RayBiotech), respectively. CT was performed with a RmCT2 system (Rigaku) to evaluate the cross-sectional area of lower limb muscle, the maximum diameter of which was quantified with the system software. The sequences of the PCR primers (forward and reverse, respectively) in Supplemental Figure 10A were 5′-CAGCTCAAGATTGTCAACCC-3′ and 5′-ATGAGGGTCAGACTCATCTG-3′.

### Cell culture, adenovirus infection, siRNA transfection, quantitative RT-PCR analysis, and immunoblot analysis.

C2C12 myoblasts described previously (4) or mouse primary myoblasts were maintained and induced to differentiate into myotubes, and they were treated with STO-609 (Calbiochem), EGTA (Wako), GsMTx-4 (Peptide Institute), Yoda1, nifedipine (Wako), or tranilast (Tokyo Chemical Industry). Adenoviral vectors for LacZ and mouse KLF15 were described previously (35). Mouse KLF15 and negative control siRNAs were obtained from Invitrogen and were delivered into cells with the use of the Lipofectamine RNAiMAX transfection reagent (Invitrogen). Isolation of total RNA and quantitative RT-PCR analysis were performed as previously described (4). Data were normalized by the amount of *36B4* mRNA. The sequences of PCR primers are provided in Supplemental Tables 2 and 3. Immunoblot analysis was performed with antibodies against STAT3 (4904, Cell Signaling Technology) and against Tyr^705^-phosphorylated STAT3 (9131, Cell Signaling Technology). Uncropped immunoblots are presented in Supplemental Figure 12.

### Histological analysis.

Skeletal muscle sections were stained with hematoxylin-eosin, and the stained muscle fibers were detected with a BZ-X710 fluorescence microscope (Keyence). The area of muscle fibers was quantified for each image with the use of ImageJ software (NIH).

### ChIP analysis.

ChIP was performed with the use of a ChIP assay kit (Merck Millipore). After cross-linking with 1% formaldehyde, nuclear lysates of C2C12 myotubes were subjected first to ultrasonic treatment to induce chromatin fragmentation and then to immunoprecipitation with antibodies against KLF15 (sc-271675, Santa Cruz Biotechnology) or negative control IgG. Target and nontarget (*Gapdh*) regions of genomic DNA in the input (positive control) and immunoprecipitated samples were amplified by quantitative RT-PCR analysis. The sequences of the PCR primers (forward and reverse, respectively) were 5′-CCATTAGAAACAACTGGTCCTG-3′ and 5′-TCATACAAAGACACACACTCCC-3′ for the mouse *Il6* promoter and 5′-CCCCACCATCCGGGTTCCTA-3′ and 5′-GATGCGGCCGTCTCTGGAAC-3′ for mouse *Gapdh*.

### DNA microarray analysis.

Total RNA extracted from gastrocnemius muscle of WT or M-KLF15KO mice at 3 days after cast immobilization or of corresponding control animals was subjected to hybridization with an Affymetrix Mouse Gene 2.0 ST Array. The data were analyzed with DAVID Bioinformatics Resources 6.8.

### Two-photon imaging.

Two-photon images were acquired from tibialis anterior muscle with a laser scanning system (LSM 7 MP, Carl Zeiss) equipped with 2 types of water-immersion objective lens (×10 and ×20, with numerical apertures of 0.5 and 1.0, respectively; Carl Zeiss) and a Ti:sapphire laser (Mai Tai HP, Spectra-Physics) operating at a wavelength of 950 nm (36, 37). Continuous 4000-frame Ca^2+^ imaging was repeated for each imaging field. The imaged fields were 848.54 by 848.54 μm (original scan) or 425.1 by 425.1 μm (×2.0 digital zoom). The pixel size was 1.657 or 0.83 μm (×2.0 digital zoom), and the frame duration was 968 ms. FRET imaging of tibialis anterior from the surface to a maximum depth of 250 μm (pixel size, 0.83 μm; frame duration, 3.87 seconds; depth interval, 2 μm) was recorded at an excitation wavelength of 830 nm (38). Fluorescence was separated by a 509-nm dichroic mirror with 460- to 500-nm (cyan channel: for CFP fluorescence detection) and 520- to 560-nm (yellow channel: for YFP fluorescence detection) emission filters.

### Patch-clamp recording.

Whole-cell patch-clamp recording was performed with a standard bath solution containing 140 mM NaCl, 5 mM KCl, 2 mM MgCl_2_, 2 mM CaCl_2_, 10 mM HEPES, and 10 mM glucose at pH 7.4 (39). Reversal potential was measured with the use of voltage ramps (–100 to +100 mV in each 5-second interval). The pipette solution for whole-cell recordings contained 120 mM potassium gluconate, 20 mM KCl, 0.5 mM EGTA, 2 mM ATP (Mg^2+^ salt), 2 mM GTP (potassium salt), and 10 mM HEPES at pH 7.4. Whole-cell recording data were sampled at 10 kHz and filtered at 5 kHz for analysis (Axon 200B amplifier with pCLAMP software; Axon Instruments). For recording of Piezo1-mediated currents, bath solution alone or containing GsMTx-4 was applied to C2C12 myotubes and the amplitude of the flow-induced currents was quantified.

### Human skeletal muscle samples.

Skeletal muscle samples were obtained from 24 individuals who underwent fixation surgery for a bone fracture of the arm or leg an average of 6.9 ± 3.5 days after cast fixation of the limb (IM group). Samples were also obtained from 27 control participants who underwent implant-removal surgery 6 to 12 months after fixation surgery. Blood samples were also obtained before surgery. The participants were recruited between July 2018 and December 2021. Given that age and biopsy site were significantly different between the IM and control groups, we matched these 2 parameters on the basis of the logit of the propensity score with the use of the nearest-neighbor matching method without replacement at a caliper width of SD × 0.25. Thus, we extracted 33 participants (15 and 18 for the IM and control groups, respectively) and compared the expression of various mRNAs. Their clinical characteristics after matching are summarized in Supplemental Table 1.

### Data availability.

The microarray data have been deposited in the NCBI Gene Expression Omnibus (GEO) under accession number GSE172501.

### Statistics.

Quantitative data are presented as mean ± SEM unless otherwise indicated. For the box-and-whisker plots (Figures 1F, 2H, and 4B), the lines within the boxes, the bounds of the boxes, and the whiskers indicate the median, the 25th and 75th percentile, and the minimum and maximum values, respectively. The values were analyzed by the 2-tailed unpaired Student’s *t* test, 2-way analysis of variance (ANOVA) with Bonferroni’s post hoc test, Mann-Whitney *U* test, Spearman’s rank correlation test, or χ^2^ test. A *P* value of less than 0.05 was considered statistically significant.

### Study approval.

All animal experiments were approved by the animal experimentation committee of Kobe University Graduate School of Medicine (approval no. P171012). All human experiments were approved by the medical ethics committee of Kobe University Graduate School of Medicine (approval no. 180059), and all participants provided written informed consent.

## Author contributions

Y Hirata, KN, and WO conceived the study and analyzed the data. KU and TH contributed to animal and cell experiments. DK, YT, and HW contributed to 2-photon imaging. TN, TF, KO, and RK contributed to analysis of human skeletal muscle. Y Hara and TA contributed to the generation of genetically engineered mice. KS contributed to whole-cell patch-clamp analysis. Y Hirata, KN, and WO wrote the manuscript.

## Supplementary Material

Supplemental data

## Version 1 03/15/2022

In-Press Preview

## Version 2 05/16/2022

Electronic publication
