# Supplementary material for: A Piezo1/KLF15/IL-6 axis mediates immobilization-induced muscle atrophy
Source: J Clin Invest. 2022 May 16;132(10):e154611. doi: 10.1172/JCI154611 (PMC9159676; doi:10.1172/JCI154611)
Supplement: Supplemental data [file jci-132-154611-s008.pdf]

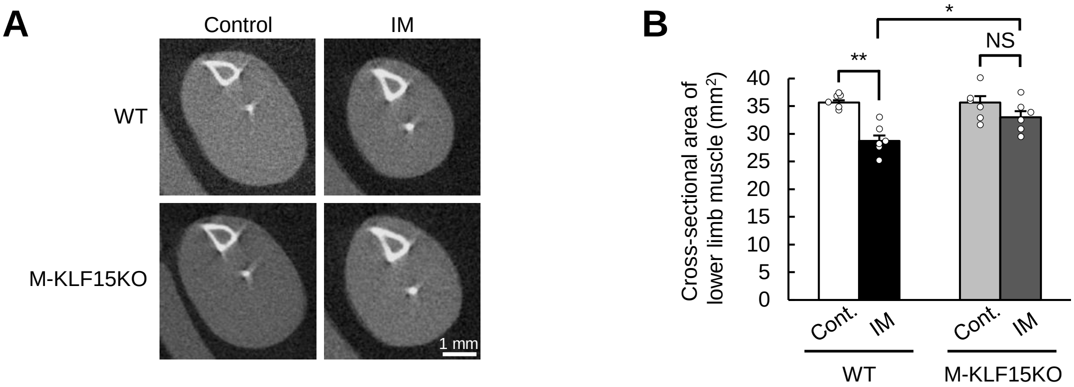

Supplemental Figure 1. Immobilization does not induce muscle atrophy in M-KLF15KO mice. Representative CT images (**A**) and quantitation of the cross-sectional area (**B**) of lower limb muscle for WT or M-KLF15KO mice subjected to cast immobilization for 3 days or for corresponding control mice (n = 6 mice) are shown. Scale bar, 1 mm. Quantitative data are means ± SEM. *P < 0.05, **P < 0.01, NS (two-way ANOVA with Bonferroni's post hoc test).

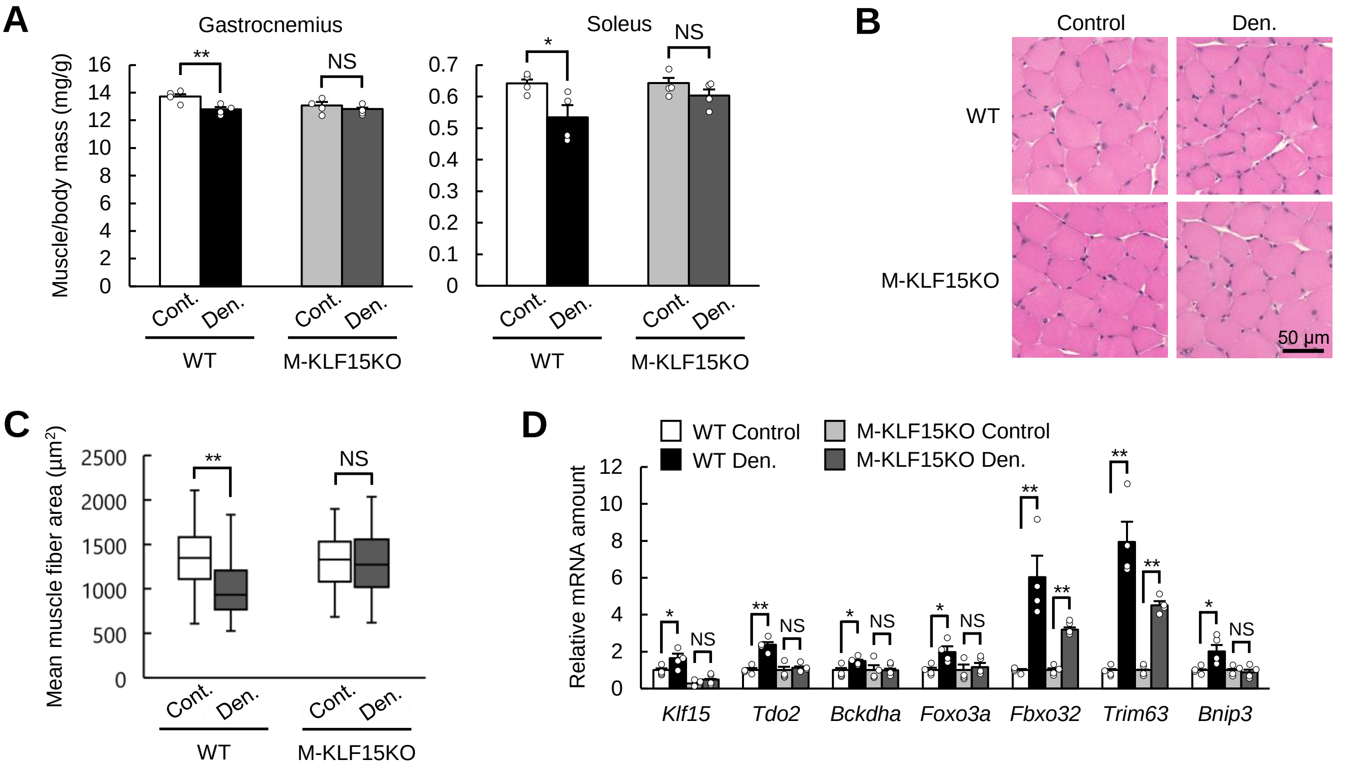

Supplemental Figure 2. Denervation-induced muscle atrophy is prevented in M-KLF15KO mice. Ratio of muscle mass to body mass (**A**), histological determination of muscle fiber area in soleus (**B**, **C**), and atrophy-related gene expression in gastrocnemius (**D**) are shown for WT or M-KLF15KO mice subjected to bilateral hind limb denervation (Den.) 2 days previously or for corresponding control mice (n = 4 mice). Scale bar in **B**, 50 μm. The area of 800 fibers pooled from four mice was measured for each condition in **C**. Quantitative data are means ± SEM (**A**, **D**) or medians (**C**). $*P < 0.05$, $**P < 0.01$, NS (two-way ANOVA with Bonferroni's post hoc test).

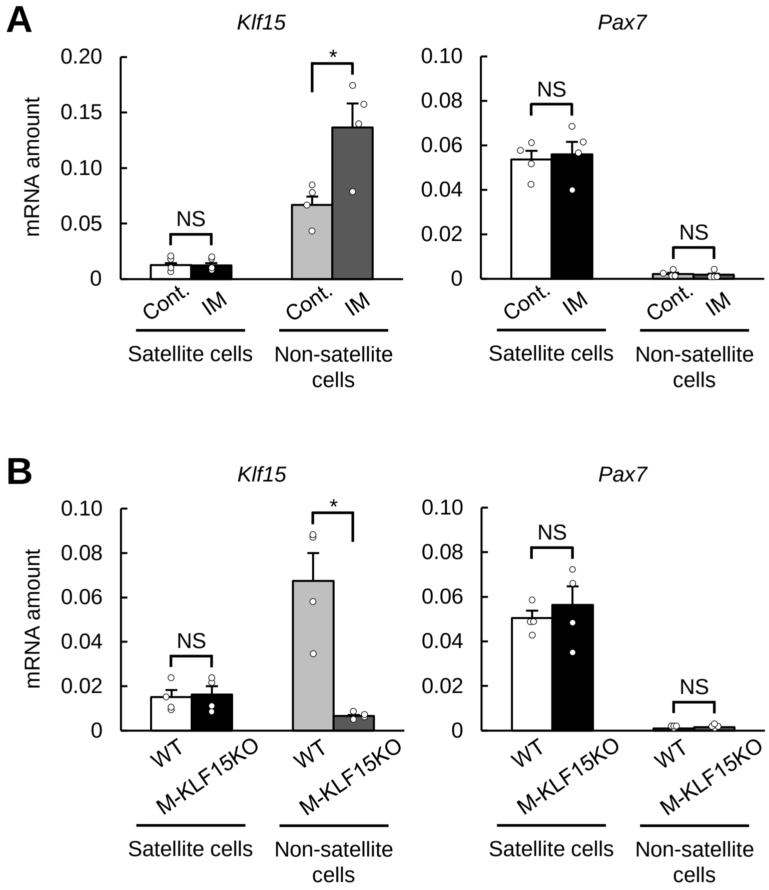

Supplemental Figure 3. Expression of *Klf15* in the satellite cell and the non-satellite cell fractions of immobilized mice or M-KLF15KO mice. A, **B**, Quantitative RT-PCR analysis of *Klf15* and *Pax7* mRNAs in the satellite cell and the non-satellite cell fractions of control or cast-immobilized mice (n = 4 mice) (**A**) or of WT or M-KLF15KO mice (n = 4 mice) (**B**). All quantitative data are means ± SEM. *$P < 0.05$, NS (two-way ANOVA with Bonferroni's post hoc test).

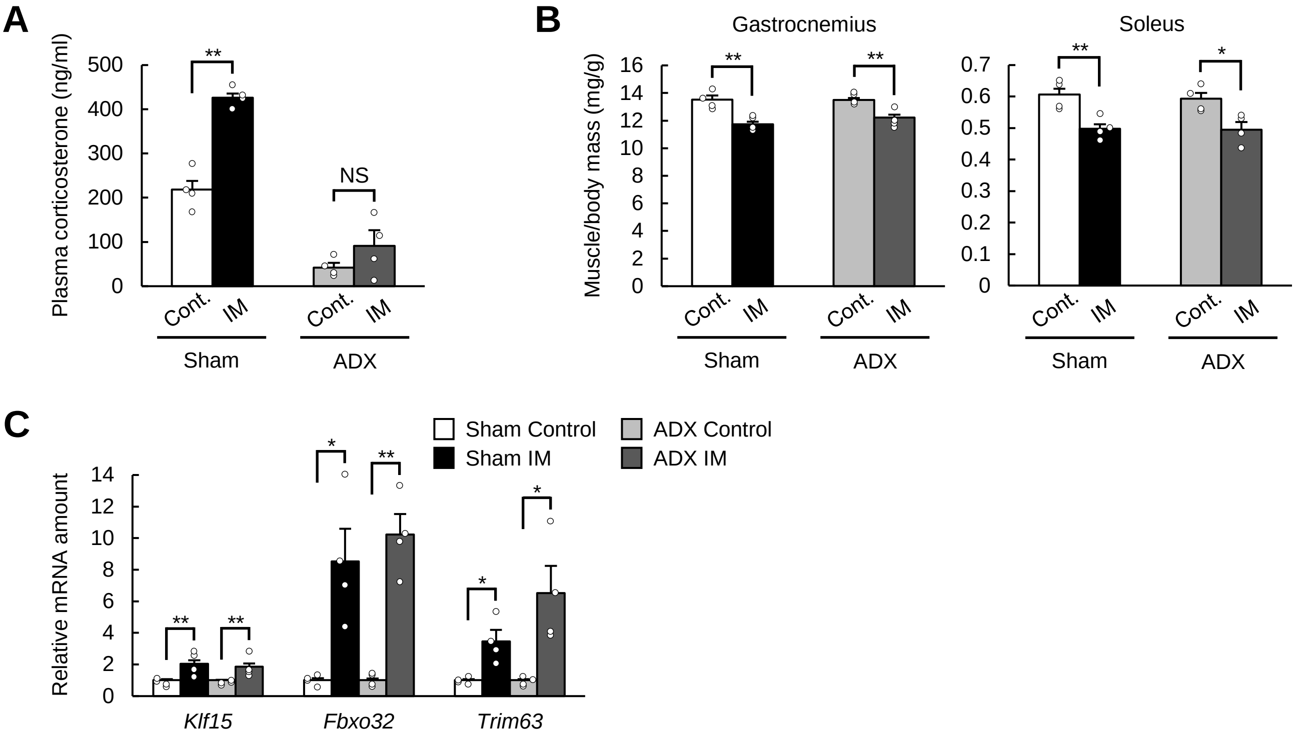

Supplemental Figure 4. Effect of adrenalectomy on immobilization-induced muscle atrophy. The plasma corticosterone level (**A**), the ratio of muscle mass to body mass (**B**), and atrophy-related gene expression in gastrocnemius (**C**) are shown for adrenalectomized (ADX) or sham-operated mice subjected to cast immobilization for 3 days or for corresponding control animals (n = 4 mice). All quantitative data are means ± SEM. *P < 0.05, **P < 0.01, NS (two-way ANOVA with Bonferroni's post hoc test).

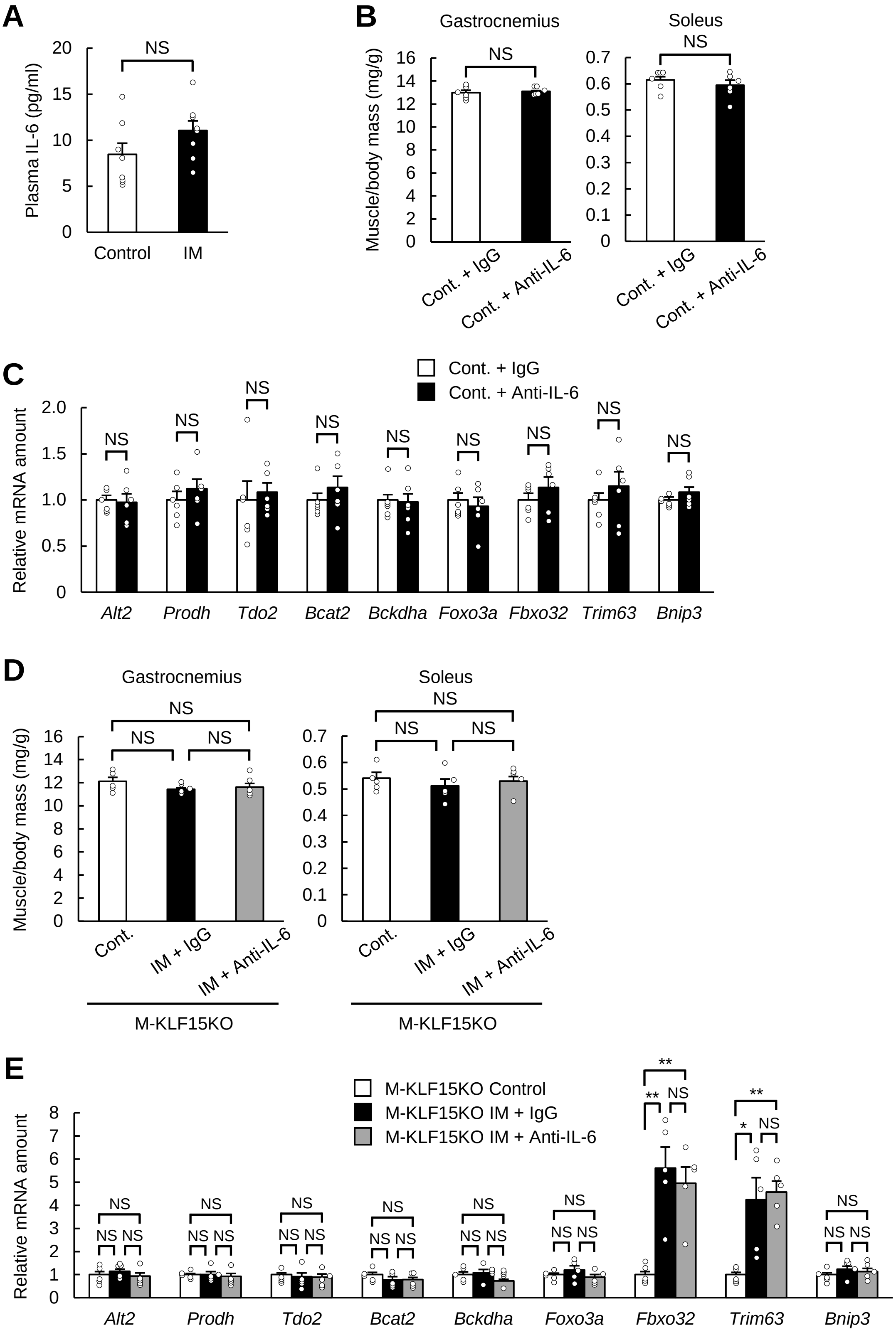

Supplemental Figure 5. Effect of neutralizing antibodies to IL-6 on skeletal muscle of control mice or immobilized M-KLF15KO mice. A, Plasma concentration of IL-6 in WT mice subjected to cast immobilization of the hind limbs for 3 days or in corresponding control mice (*n* = 8 mice). **B**, **C**, Ratio of muscle mass to body mass (*n* = 6 mice) (**B**) and atrophy-

related gene expression in gastrocnemius (*n* = 6 mice) (**C**) are shown for control mice subjected to intraperitoneal injection of neutralizing antibodies to IL-6 (0.1 mg/body) or control IgG. **D**, **E**, Ratio of muscle mass to body mass (*n* = 5 mice) (**D**) and atrophy-related gene expression in gastrocnemius (*n* = 5 mice) (**E**) are shown for control or cast-immobilized M-KLF15KO mice subjected to intraperitoneal injection of neutralizing antibodies to IL-6 (0.1 mg/body) or control IgG at the onset of limb immobilization. All quantitative data are means ± SEM. *$P < 0.05$, **$P < 0.01$, NS by the unpaired *t* test (**A–C**) or two-way ANOVA with Bonferroni's post hoc test (**D**, **E**).

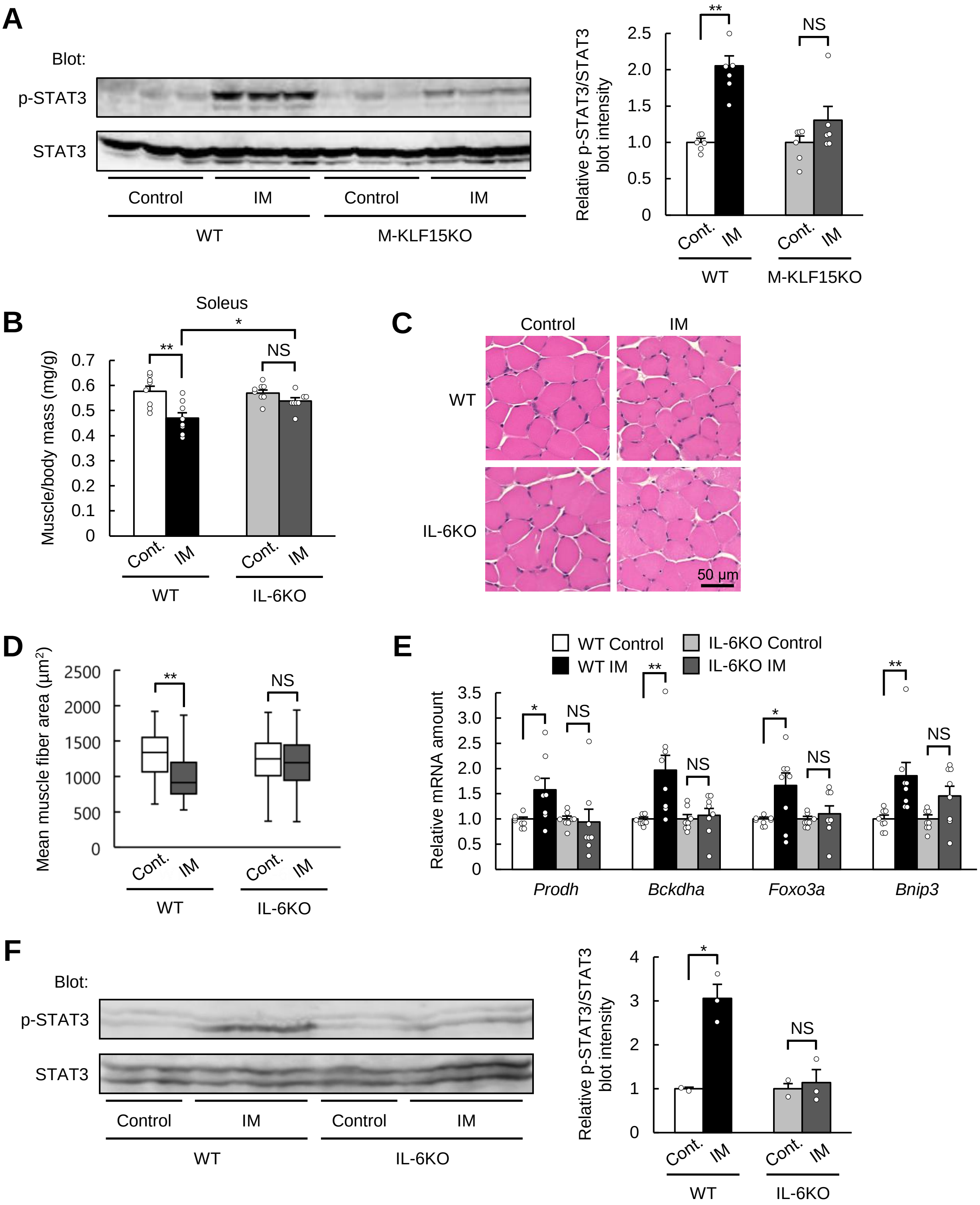

Supplemental Figure 6. Immobilization does not induce muscle atrophy in IL-6KO mice. A, Immunoblot analysis of the abundance and phosphorylation of STAT3 in gastrocnemius of WT or M-KLF15KO mice subjected to hind limb immobilization for 3 days or of corresponding control mice (*n* = 6 mice). **B**–**F**, Ratio of muscle mass to body mass (*n* = 8 mice) (**B**), histological determination of muscle fiber area in soleus (**C**, **D**), atrophy-related gene expression in gastrocnemius (*n* = 8 mice) (**E**), and immunoblot analysis of the abundance and phosphorylation of STAT3 in gastrocnemius (control, *n* = 2; IM, *n* = 3 mice)

(**F**) for WT or IL-6KO mice subjected to hind limb immobilization for 3 days or for corresponding control mice. Scale bar in **C**, 50 μm. The area of 800 fibers pooled from four mice was measured for each condition in **D**. Quantitative data are means ± SEM (**A**, **B**, **E**, **F**) or medians (**D**). **P* < 0.05, ***P* < 0.01, NS (two-way ANOVA with Bonferroni's post hoc test).

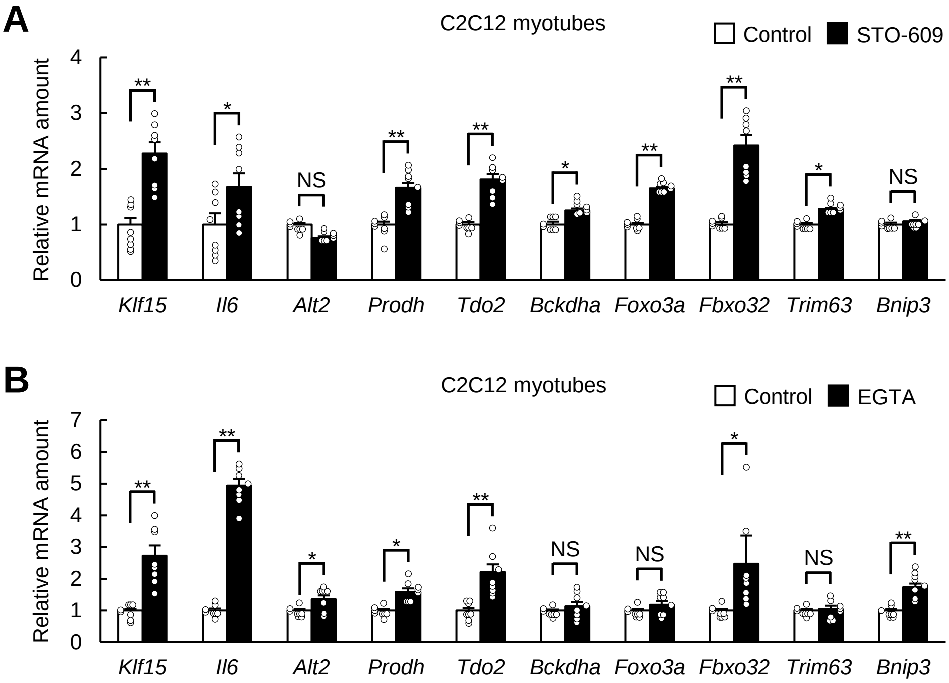

Supplemental Figure 7. A decrease in $[Ca^{2+}]_i$ to below the basal level is associated with muscle atrophy. A, **B**, Quantitative RT-PCR analysis of the expression of atrophy-related genes including *Klf15* and *Il6* in C2C12 myotubes exposed to 2 μM STO-609 or vehicle (Control) for 48 h (*n* = 8 independent experiments) (**A**) or to 0.1 mM EGTA or vehicle (Control) for 3 h (*n* = 8 independent experiments) (**B**). All quantitative data are means ± SEM. *P < 0.05, **P < 0.01, NS (unpaired *t* test).

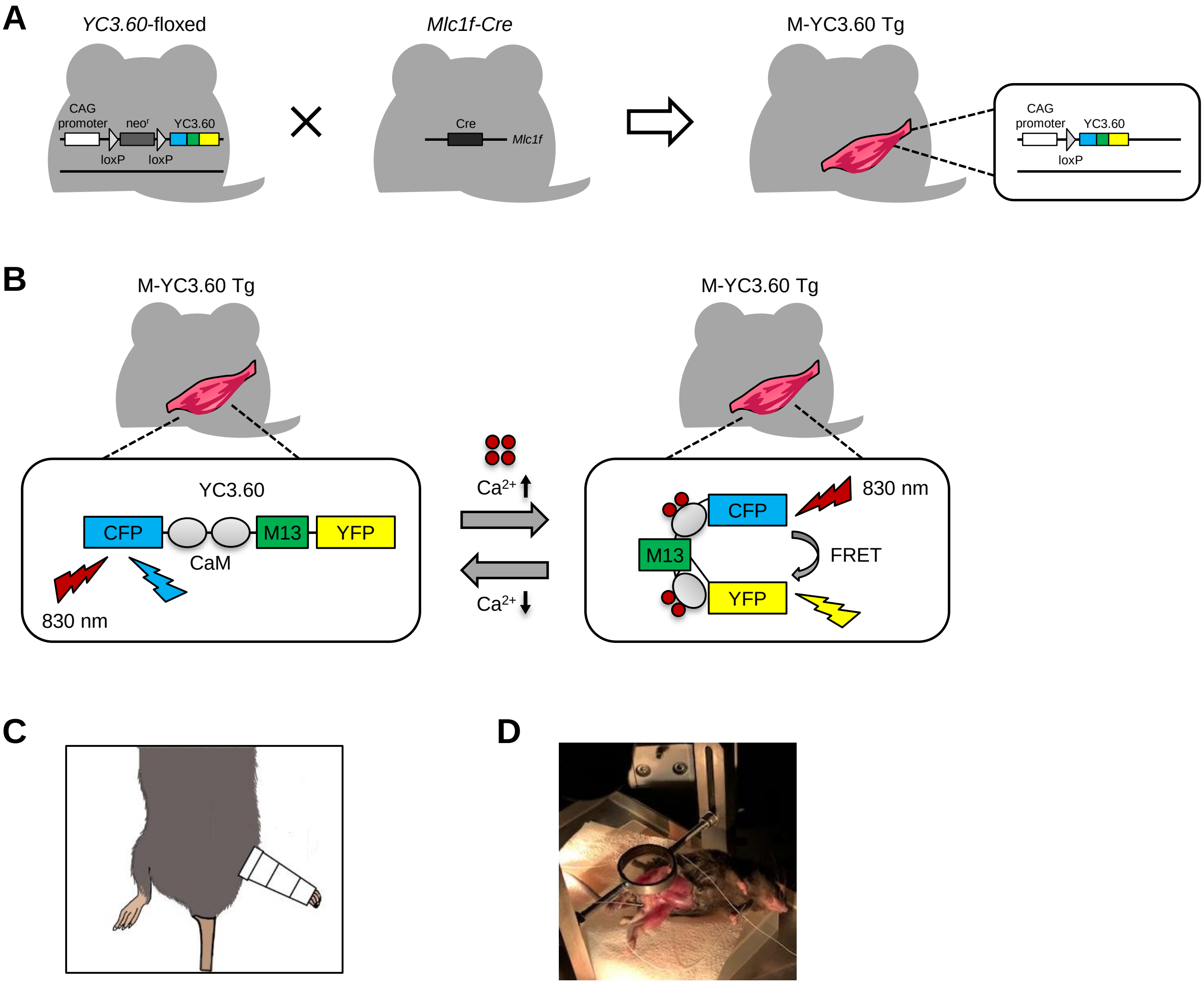

Supplemental Figure 8. Intravital Ca^{2+} imaging of skeletal muscle–specific YC3.60 transgenic mice. A–C, Schematic representation of the generation of M-YC3.60 Tg mice by crossing of *YC3.60*-floxed mice and *Mlc1f-Cre* mice (**A**), of the principle of the YC3.60 FRET sensor (**B**), and of unilateral cast immobilization of the hind limb (**C**). **D**, Photograph of the setup for two-photon microscopy.

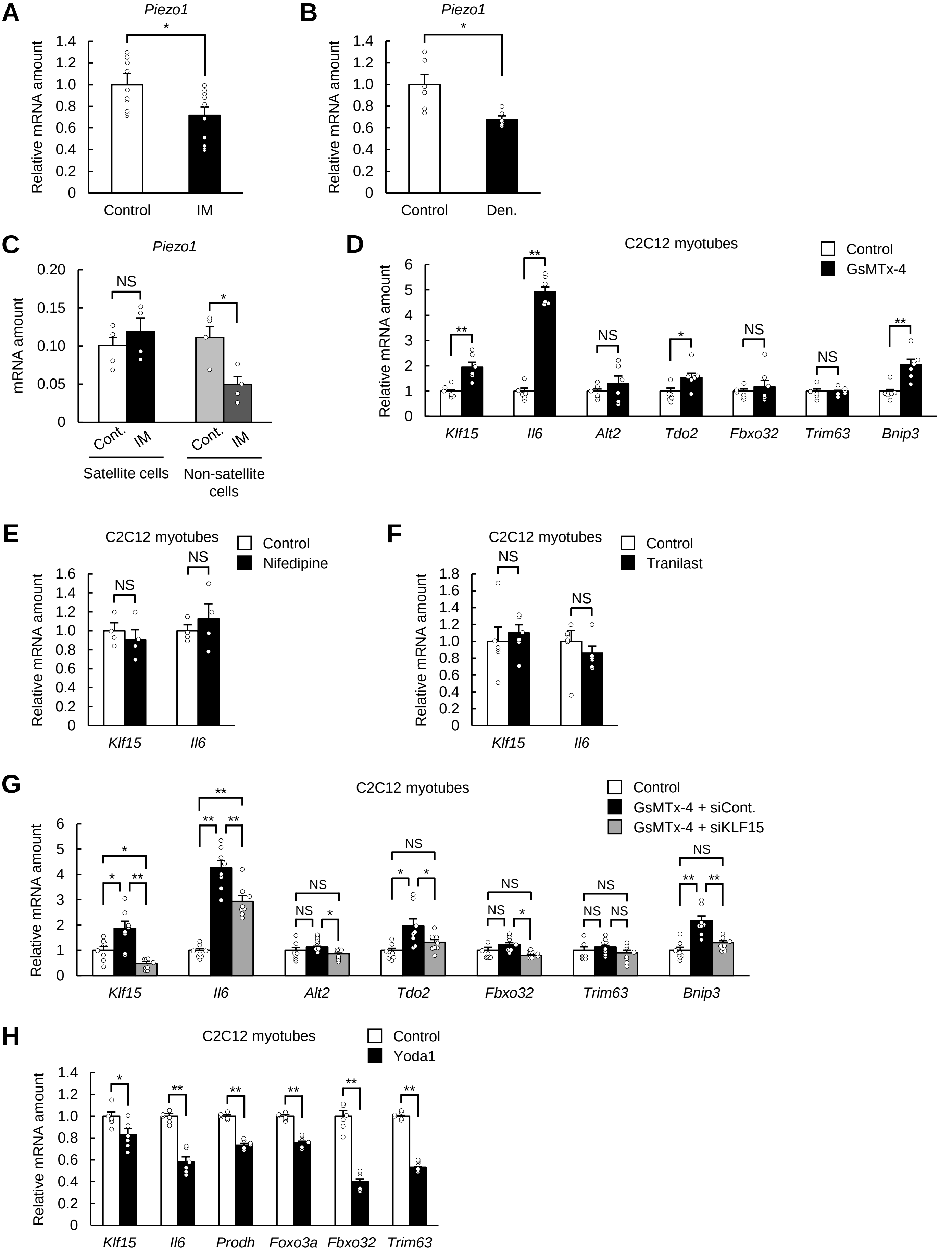

A
Piezo1
Relative mRNA amount
Control
IM
B
Piezo1
Relative mRNA amount
Control
Den.
C
Piezo1
mRNA amount
NS
Cont.
IM
Satellite cells
Non-satellite cells
D
C2C12 myotubes
Control
GsMTx-4
Relative mRNA amount
Klf15
Il6
Alt2
Tdo2
Fbxo32
Trim63
Bnip3
E
C2C12 myotubes
Control
Nifedipine
F
C2C12 myotubes
Control
Tranilast
G
C2C12 myotubes
Control
GsMTx-4 + siCont.
GsMTx-4 + siKLF15
H
C2C12 myotubes
Control
Yoda1
Prodh
Foxo3a

Supplemental Figure 9. Expression of *Piezo1* in skeletal muscle of immobilized mice and effects of Piezo1 channel inhibitor or activator. A, **B,** Quantitative RT-PCR analysis of *Piezo1* mRNA in gastrocnemius of control or cast-immobilized mice (n = 10 mice) (**A**) or of control or denervated mice (n = 6 mice) (**B**). **C**, Quantitative RT-PCR analysis of *Piezo1* mRNA in the satellite cell and the non-satellite cell fractions of control or cast-immobilized mice (n = 4 mice). **D**, Quantitative RT-PCR analysis of the expression of atrophy-related genes including *Klf15* and *Il6* in C2C12 myotubes exposed to 50 μM GsMTx-4 or vehicle (Control) for 6 h (n = 6 independent experiments). **E**, **F**, Quantitative RT-PCR analysis of *Klf15* and *Il6* mRNAs in C2C12 myotubes exposed to 10 μM nifedipine or vehicle (Control) for 6 h (n = 4 independent experiments) (**E**) or to 75 μM tranilast or vehicle (Control) for 6 h (n = 6 independent experiments) (**F**). **G**, Quantitative RT-PCR analysis of the expression of atrophy-related genes including *Klf15* and *Il6* in C2C12 myotubes exposed to vehicle (Control) or 50 μM GsMTx-4 as well as transfected with control (siCont) or KLF15 (siKLF15) siRNAs for 6 h (n = 8 independent experiments). **H**, Quantitative RT-PCR analysis of the expression of atrophy-related genes including *Klf15* and *Il6* in C2C12 myotubes exposed to 50 μM Yoda1 or vehicle (Control) for 6 h (n = 6 independent experiments). All quantitative data are means ± SEM. *P < 0.05, **P < 0.01, NS by the unpaired t test (**A**, **B**, **D**–**F**, **H**) or two-way ANOVA with Bonferroni's post hoc test (**C**, **G**).

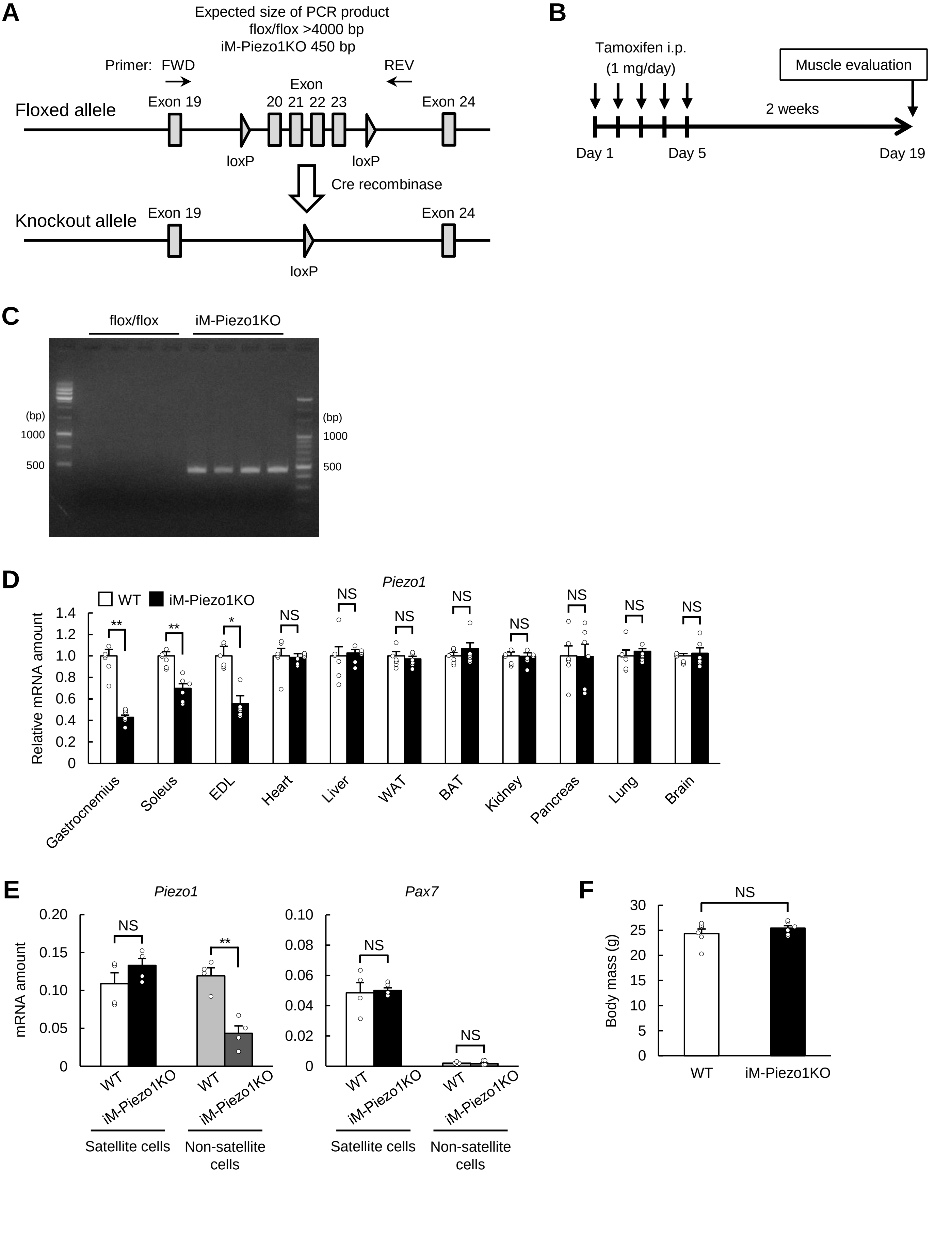
A
Expected size of PCR product
flox/flox >4000 bp
iM-Piezo1KO 450 bp
Primer: FWD
REV
Exon
20 21 22 23
Floxed allele
Exon 19
Exon 24
loxP
loxP
Cre recombinase
Knockout allele
Exon 19
Exon 24
loxP
B
Tamoxifen i.p.
(1 mg/day)
Muscle evaluation
2 weeks
Day 1
Day 5
Day 19
C
flox/flox
iM-Piezo1KO
(bp)
1000
500
D
Piezo1
WT
iM-Piezo1KO
Relative mRNA amount
**
*
NS
Gastrocnemius
Soleus
EDL
Heart
Liver
WAT
BAT
Kidney
Pancreas
Lung
Brain
E
Piezo1
Pax7
mRNA amount
WT
iM-Piezo1KO
Satellite cells
Non-satellite cells
F
Body mass (g)
WT
iM-Piezo1KO

Supplemental Figure 10. Generation of tamoxifen-inducible skeletal muscle–specific Piezo1 KO (iM-Piezo1KO) mice. A, **B**, Schematic representation of the floxed and KO alleles of mouse *Piezo1* showing the positions of PCR primers (**A**) as well as of the protocol for intraperitoneal (i.p.) injection of tamoxifen and muscle evaluation in iM-Piezo1KO mice (**B**). **C**, Gel electrophoresis of PCR products for genomic DNA isolated from gastrocnemius of WT (flox/flox) or iM-Piezo1KO mice at 2 weeks after intraperitoneal injection of tamoxifen (n = 4 mice). The outer lanes contain molecular size markers. **D**, Quantitative RT-PCR analysis of the tissue distribution of *Piezo1* mRNA in WT or iM-Piezo1KO mice at 2 weeks after intraperitoneal injection of tamoxifen (n = 6 mice). EDL, extensor digitorum longus; WAT and BAT, white and brown adipose tissue, respectively. **E**, Quantitative RT-PCR analysis of *Piezo1* and *Pax7* mRNAs in the satellite cell and the non-satellite cell fractions of WT or iM-Piezo1KO mice at 2 weeks after intraperitoneal injection of tamoxifen (n = 4 mice). **F**, Body mass of WT and iM-Piezo1KO mice at 2 weeks after intraperitoneal injection of tamoxifen (n = 6 mice). All quantitative data are means ± SEM. *P < 0.05, **P < 0.01, NS by the unpaired t test (**D**, **F**) or two-way ANOVA with Bonferroni's post hoc test (**E**).

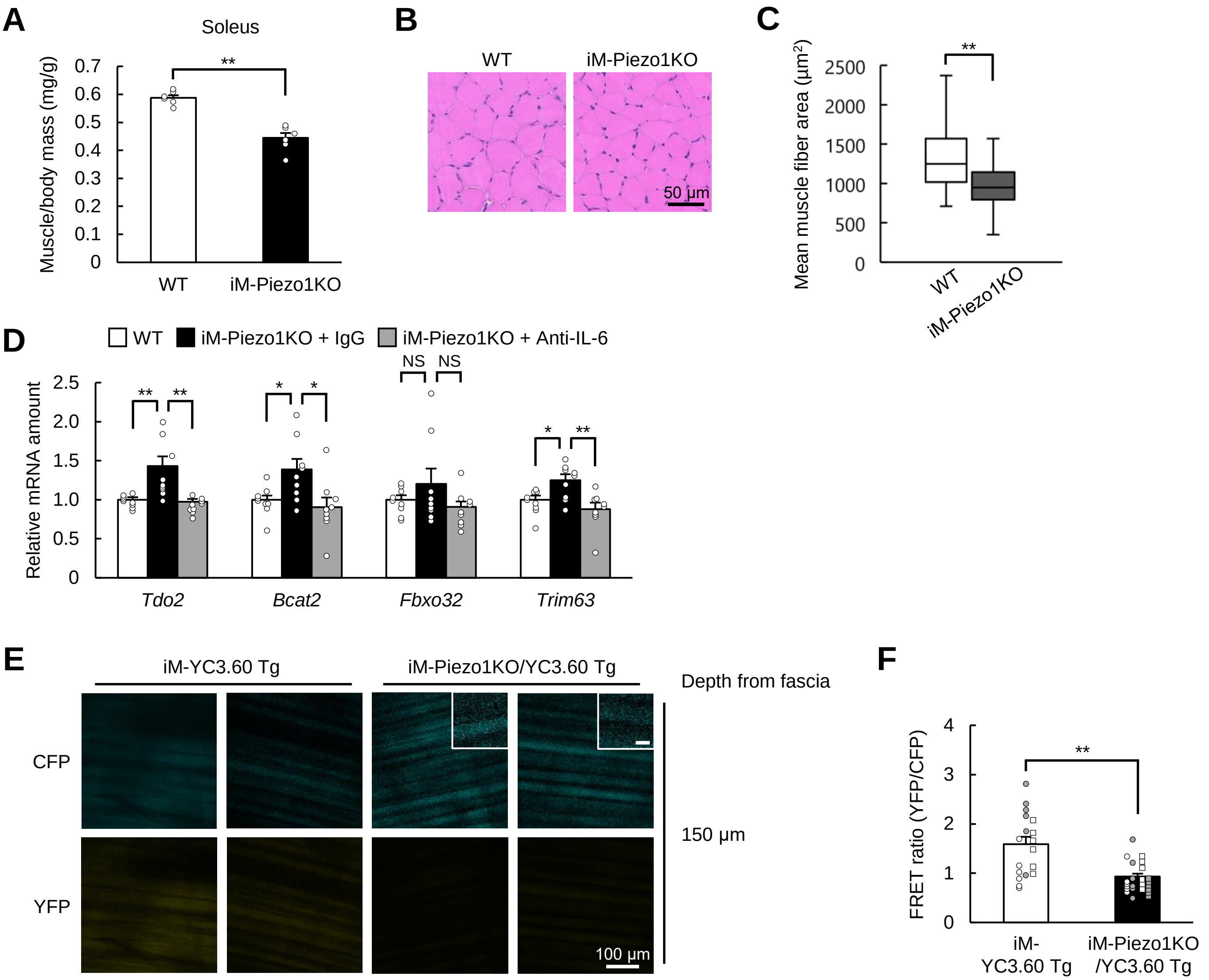

Supplemental Figure 11. The phenotype of iM-Piezo1KO mice. A–C, Ratio of soleus muscle mass to body mass (*n* = 6 mice) (**A**), and histological determination of muscle fiber area in soleus (**B**, **C**) for WT or tamoxifen-treated iM-Piezo1KO mice. The area of 800 fibers pooled from four mice was measured for each condition in **C**. Scale bars, 50 μm. **D**, Quantitative RT-PCR analysis of the expression of atrophy-related genes in gastrocnemius of WT or iM-Piezo1KO mice subjected to intraperitoneal injection of neutralizing antibodies to IL-6 (0.1 mg/body) or control IgG at the onset of tamoxifen treatment (*n* = 9 mice). **E**, **F**, Intravital Ca^{2+} imaging of iM-Piezo1KO/YC3.60 Tg mice. Representative two-photon images of CFP and YFP fluorescence at a depth of 150 μm from the fascia of the tibialis anterior muscle for tamoxifen-treated iM-YC3.60 Tg mice or iM-Piezo1KO/YC3.60 Tg mice are shown in **E**. Scale bars, 100 μm (main panels) or 20 μm (insets). Quantitation of the FRET ratio in areas of six fibers for each of three (iM-YC3.60 Tg) or four (iM-Piezo1KO/YC3.60 Tg) hind limbs is shown in **F**, with white or gray circles or squares indicating the values obtained from individual hind limbs. Quantitative data are means ± SEM (**A**, **D**, **F**) or medians (**C**). *P < 0.05, **P < 0.01, NS by the unpaired t test (**A**, **C**, **F**) or two-way ANOVA with Bonferroni's post hoc test (**D**).

Figure 2J

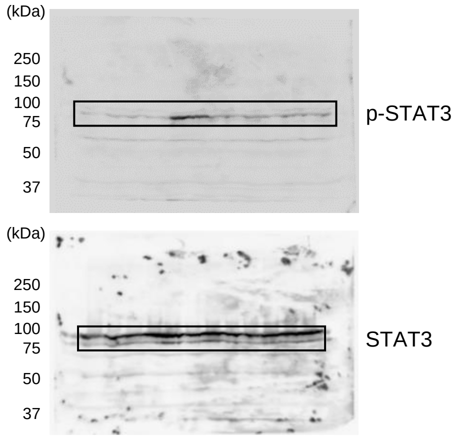

Figure 6E

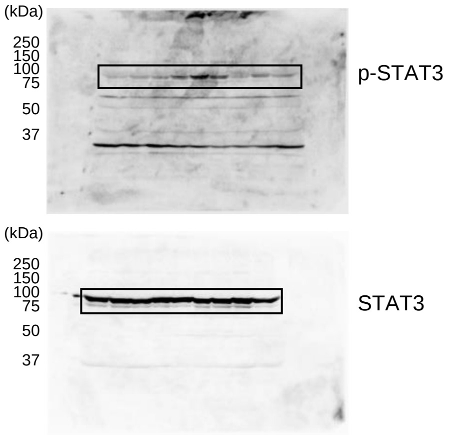

Supplemental Figure 6A

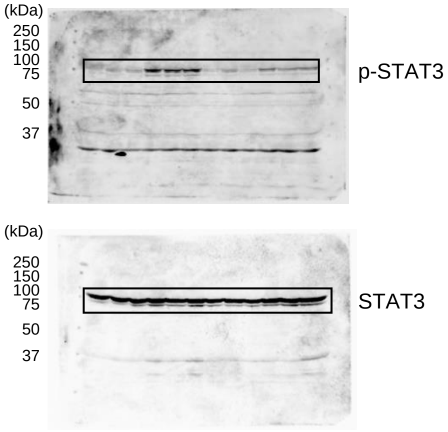

Supplemental Figure 6F

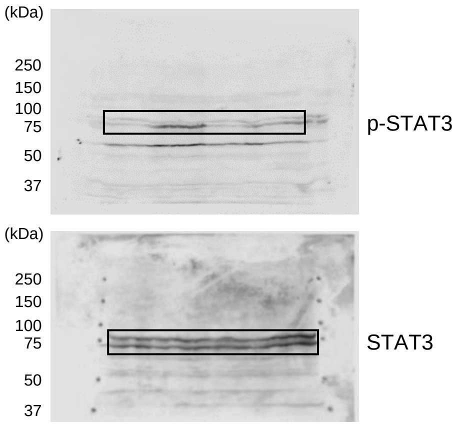

Supplemental Figure 12. Uncropped immunoblot images. Black boxes contain the areas shown in the indicated figures.

Characteristic	Control group	IM group	*P* value
N	18	15	
Age (years)	63.9 ± 15.0	69.1 ± 11.7	0.29
Sex (male/female)	6/12	5/10	1.00
Body mass index (kg/m^2)	23.3 ± 4.2	24.1 ± 5.1	0.63
Duration of immobilization (days)		8.3 ± 3.1	
Site of biopsy (upper/lower limb)	10/8	8/7	0.90
Serum creatinine (mg/dl)	0.80 ± 0.15	1.44 ± 2.22	0.25
Serum creatine kinase (U/l)	126.3 ± 58.0	183.5 ± 288.6	0.46
Hemoglobin A_{1c} (%)	5.8 ± 0.6	6.1 ± 0.9	0.44

Supplemental Table 1. Clinical characteristics of control and immobilized human subjects. Data for continuous variables are means ± SD. The *P* values were determined with the chi-square test (sex distribution and site of biopsy) or unpaired *t* test (other parameters).

Mouse gene	Primers (5' → 3')
36b4	FWD: GAGGAATCAGATGAGGATATGGGA<br>REV: AAGCAGGCTGACTTGGTTGC
Adgre1	FWD: TGCCTCCCTGACTTTCAAAT<br>REV: TGGCATTGCTGTATCTGCTC
Alt2	FWD: CAGACCCAGACAACATTTACCTG<br>REV: CGCGGAGTACAAGGGATACTG
Bcat2	FWD: TGGAGTGGAATAACAAGGCTG<br>REV: GTCTCCACCTTTGTATGCTTTC
Bckdha	FWD: GCAGCCTATGCTGCCAAGC<br>REV: GATGGCATAGCCATTGTTCCG
Bnip3	FWD: TTCCACTAGCACCTTCTGATGA<br>REV: GAACACCGCATTTACAGAACAA
Fbxo32	FWD: GCAAACACTGCCACATTCTCTC<br>REV: CTTGAGGGGAAAGTGAGACG
Foxo3a	FWD: CAGGCTCCTCACTGTATTCAGCTA<br>REV: CATTGAACATGTCCAGGTCCAA
Il1b	FWD: GCTGAAAGCTCTCCACCTCA<br>REV: AGGCCACAGGTATTTTGTCG
Il6	FWD: CAAAGCCAGAGTCCTTCAGAG<br>REV: GCCACTCCTTCTGTGACTCC
Klf15	FWD: ACCGAAATGCTCAGTGGGTTACCTA<br>REV: GGAACAGAAGGCTTGCGAGTCA
Mcp1	FWD: AGGTGTCCCAAAGAAGCTGTA<br>REV: ATGTCTGGACCCATTCCTTCT
Piezo1	FWD: ATCCTGCTGTATGGGCTGAC<br>REV: AAGGGTAGCGTGTGTGTTCC
Prodh	FWD: TCATCAGTGCCCGCACCTAC<br>REV: TGCAGTGAGCTTAATGGCTGAGA
Tdo2	FWD: TGCTCAAGGTGATAGCTCGGA<br>REV: AGGAGCTTGAAGATGACCACCA
Tnfa	FWD: CATCTTCTCAAAATTCGAGTGACAA<br>REV: TGGGAGTAGACAAGGTACAACCC
Trim63	FWD: GCTGGTGGAAAACATCATTGACAT<br>REV: CATCGGGTGGCTGCCTTT

Supplemental Table 2. Sequences of mouse primers for quantitative RT-PCR analysis. FWD, forward; REV, reverse.

Human gene	Primers (5’ → 3’)
36B4	FWD: TTGTGGGAGCAGACAATGTG<br>REV: TATTGGCCAGCAACATGTCC
BCAT2	FWD: TCATCGAAGTGGACAAGGAC<br>REV: TAACTTGTAGTTGCCGACCC
BNIP3	FWD: CTGAAACAGATACCCATAGCATT<br>REV: CCGACTTGACCAATCCCA
FBXO32	FWD: AAGTCTGTGCTGGTCGGGAA<br>REV: AGTGAAGGTGAGGCCTTTGAAG
FOXO3A	FWD: TTCAAGGATAAGGGCGACAGCAAC<br>REV: CTGCCAGGCCACTTGGAGAG
IL6	FWD: GGTACATCCTCGACGGCATCT<br>REV: GTGCCTCTTTGCTGCTTTCAC
KLF15	FWD: CAAAAGCAGCCACCTCAAG<br>REV: TCTTCTCGCACACAGGACAC
PIEZO1	FWD: CAATGAGGAGGCCGACTACC<br>REV: GCACTCCTGCAGCTCGATGA
PRODH	FWD: GTGTACAAGTACGTGCCCTATGG<br>REV: TCATGAGGCTGCTGTTCTCCAG
TDO2	FWD: GGTTCCTCAGGCTATCACTACC<br>REV: CAGTGTCGGGGAATCAGGT
TRIM63	FWD: GGAGCCACCTTCCTCTTGAC<br>REV: GTCAATGGCTCTCAGGGCGT

Supplemental Table 3. Sequences of human primers for quantitative RT-PCR analysis. FWD, forward; REV, reverse.
